# Supplementary figures and images for: Genomic characterisation of an entomopathogenic strain of Serratia ureilytica in the critically endangered phasmid Dryococelus australis
Source: PLoS One. 2022 Apr 20;17(4):e0265967. doi: 10.1371/journal.pone.0265967 (PMC9020675; doi:10.1371/journal.pone.0265967)

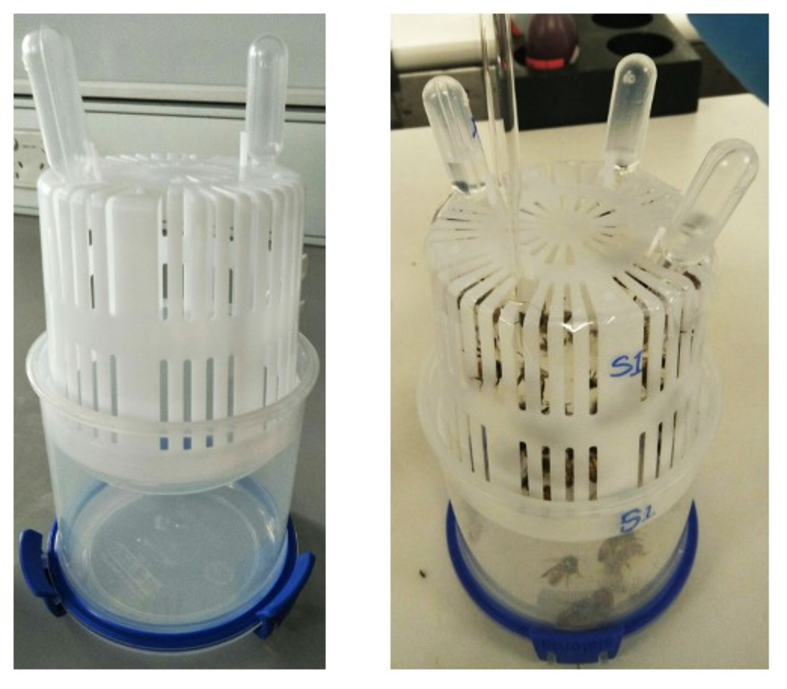

Supplement: S1 Fig — Adapted from the Sistema KLIP IT™ Utility Collection round container with strainer, 0.7 L with transfer pipette bulbs inserted to supply water and 50% sucrose solution. (TIF) [file pone.0265967.s001.tif]

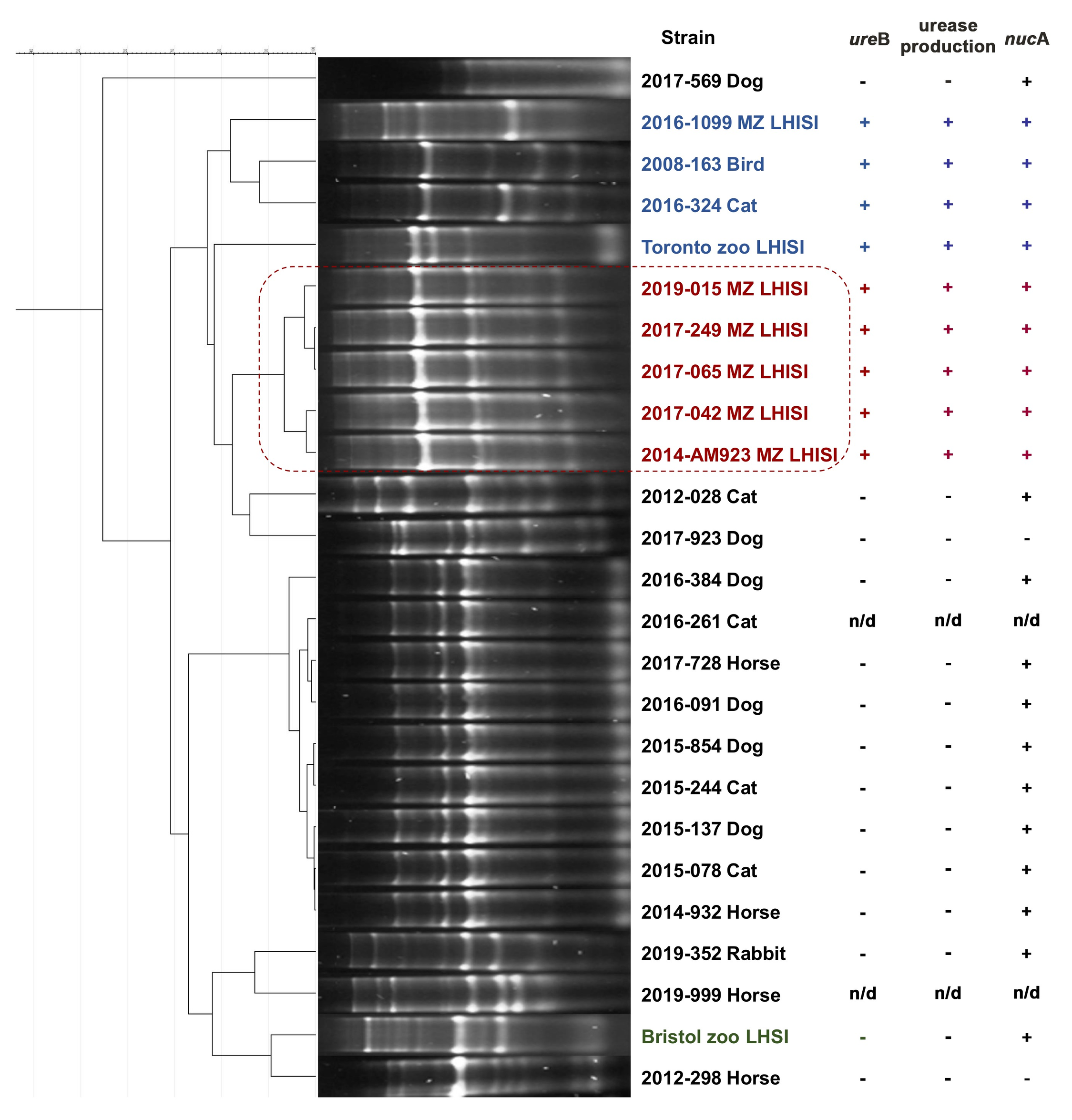

Supplement: S2 Fig — A subset of Lord Howe Island stick insect isolates from Melbourne Zoo in 2017 and 2019, indicated by the red hashed box, displayed a rep-PCR fingerprint highly similar to Serratia ureilytica AM923. Dendrogram built with GelJ using Pearson curve-based similarity coefficients with the UPGMA linkage method. Isolation year, strain number, host and results of PCR assays (ureB and nucA) and the urease production phenotypic test are indicated for each lane; n/d: no data. MZ: Lord Howe Island stick insect isolates from Melbourne Zoo. Molecular weight marker: HyperLadder™ 1kb (Meridian Biosciences). (TIF) [file pone.0265967.s002.tif]

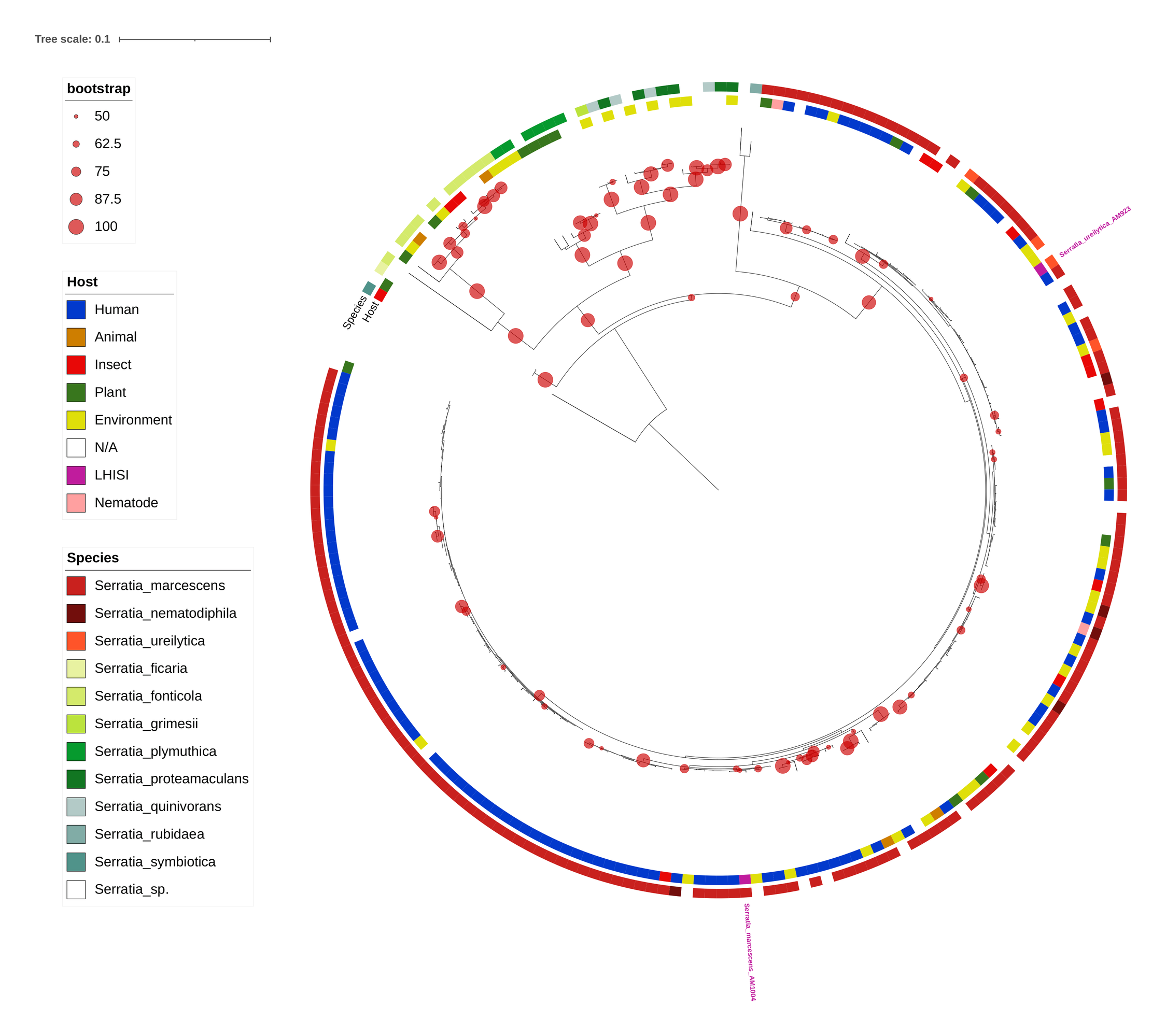

Supplement: S3 Fig — A mid-rooted tree was built from 3069 positions of 212 non-identical sequences, including 169 S. marcescens, S. nematodiphila and S. ureilytica isolates, with RAxML using a GTR + Gamma model. Bootstrap analysis was performed on 100 replicates, and support values >50% are represented by circles with diameters proportional to the value. The Melbourne Zoo isolates AM923 and AM1004 are indicated on the outside ring. The scale bars indicate the number of substitutions per site. (TIF) [file pone.0265967.s003.tif]

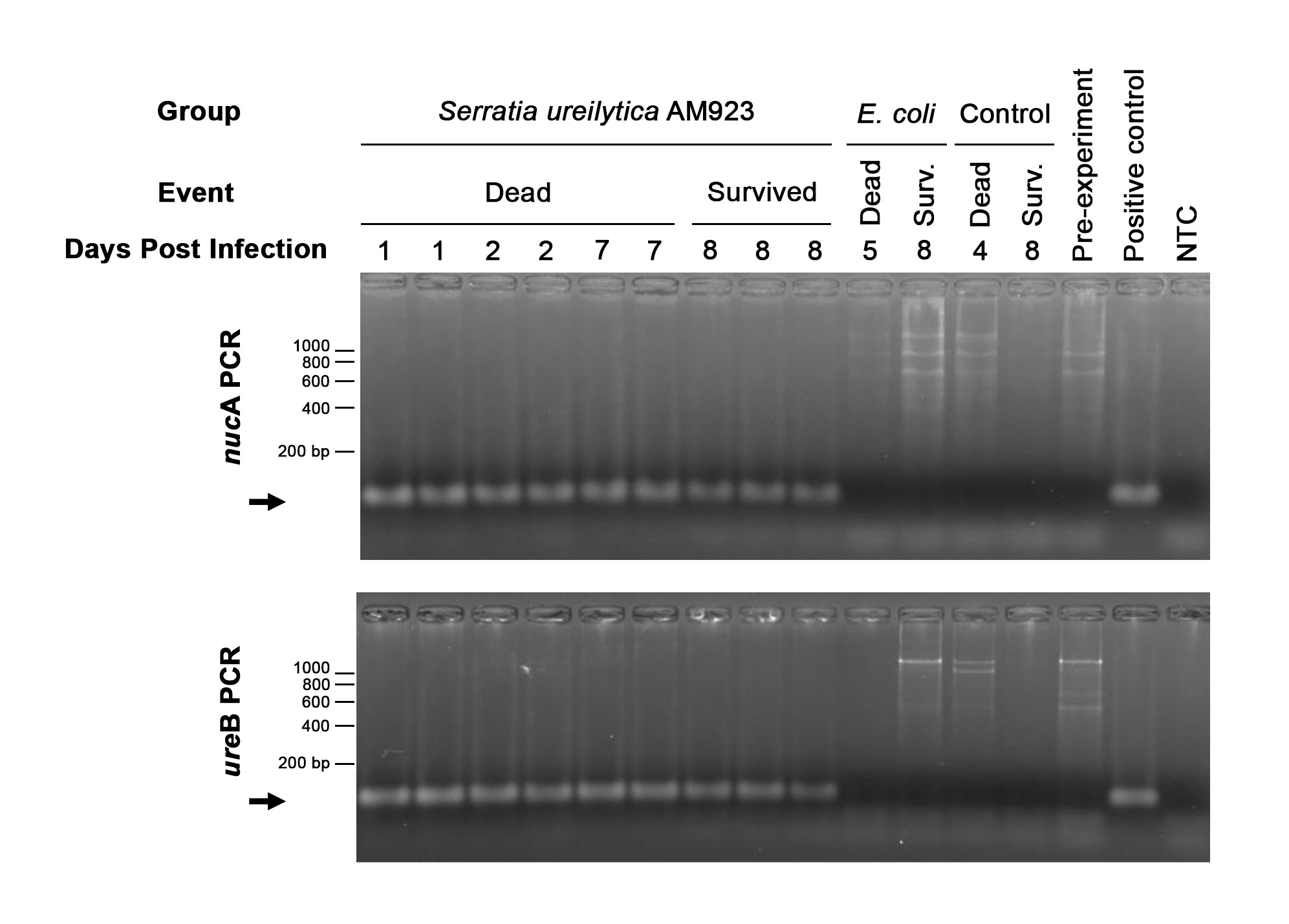

Supplement: S4 Fig — Two PCR assays were conducted to confirm detection of Serratia spp. in bees exposed to AM923, E. coli K12, or 1:1 mixture of sterile PBS and the sucrose/water mix (control). Arrows indicate the expected amplicon on the 3% agarose gel. The nature and time of the event is indicated for each bee. Dead: the individual was found dead during the experiment; Survivor: the individual was euthanised at the conclusion of the experiment; Pre-experiment: honey bee collected from the hive before inoculation; NTC: no template control. Molecular weight marker: HyperLadder™ 1kb (Meridian Biosciences). (TIF) [file pone.0265967.s004.tif]
