# Supplementary material for: Genomic characterisation of an entomopathogenic strain of Serratia ureilytica in the critically endangered phasmid Dryococelus australis
Source: PLoS One. 2022 Apr 20;17(4):e0265967. doi: 10.1371/journal.pone.0265967 (PMC9020675; doi:10.1371/journal.pone.0265967)
Supplement: S1 Table — (DOCX) [file pone.0265967.s005.docx]

**S1 Table. Details of the 131 *Serratia* spp. used in REALPHY phylogenomic analysis.**

| Genbank acc. | Species | Strain | Host | Source | Country | Year |
| --- | --- | --- | --- | --- | --- | --- |
| GCA_900187015.1 | *Serratia ficaria* | NCTC12148^a^ |  |  |  | 1981 |
| GCA_001006005.1 | *Serratia fonticola* | DSM_4576 |  | Water |  | 1979 |
| GCA_001514455.1 | *Serratia fonticola* | GS2 | Sesame | Soil | South Korea | 2015 |
| GCA_002588845.1 | *Serratia fonticola* | FDAARGOS_411 | Wildlife | Liver tissue |  |  |
| GCA_005489985.1 | *Serratia fonticola* | MS5 | Aedes aegypti |  | USA, Las Cruces | 2017 |
| GCA_011691375.1 | *Serratia fonticola* | CPSE11 |  | Codonopsis pilosula | China, Gansu | 2019 |
| GCA_013284015.1 | *Serratia fonticola* | HH13 |  |  |  | 2009 |
| GCA_900638145.1 | *Serratia fonticola* | NCTC13193 |  |  |  |  |
| GCA_000975245.1 | *Serratia liquefaciens* | HUMV_21 | Homo sapiens | Skin ulcer | Spain, Santander | 2009 |
| GCA_001559135.2 | *Serratia liquefaciens* | FDAARGOS_125 | Homo sapiens |  | USA, DC | 2013 |
| GCA_003074975.2 | *Serratia liquefaciens* | JL02 | cattle | Milk | China, Jilin | 2017 |
| GCA_006970665.1 | *Serratia liquefaciens* | FG3 | Stachytarpheta glabra |  | Brazil, Minas Gerais | 2016 |
| GCA_008364325.2 | *Serratia liquefaciens* | S1 |  | Mixed salads | Germany | 2015 |
| GCA_014495865.1 | *Serratia liquefaciens* | MT49 |  | Groundwater | USA, Oak Ridge, Tennessee | 2013 |
| GCA_016728005.1 | *Serratia liquefaciens* | FDAARGOS_1081 |  |  | Germany, Langen |  |
| GCA_000422085.1 | *Serratia liquefaciens* | ATCC_27592^a^ |  |  |  |  |
| GCA_000783915.2 | *Serratia marcescens* | FDAARGOS_65 | Homo sapiens | Endotracheal aspirate | USA | 2013 |
| GCA_001022215.1 | *Serratia marcescens* | CAV1492 | Homo sapiens | Respiratory | USA, Virginia | 2011 |
| GCA_001280365.1 | *Serratia marcescens* | RSC_14 | Solanum nigrum |  | South Korea | 2013 |
| GCA_001294565.1 | *Serratia marcescens* | SmUNAM836 | Homo sapiens | Bronchial aspirate | Mexico, Distrito Federal | 2005 |
| GCA_001417865.2 | *Serratia marcescens* | B3R3 | Zea mays |  | China, Shandong | 2011 |
| GCA_001672055.1 | *Serratia marcescens* | U36365 | Homo sapiens | Urine | India, Vellore, TN | 2015 |
| GCA_002220515.1 | *Serratia marcescens* | UMH2 | Homo sapiens | University of Michigan | USA, Michigan | 2014 |
| GCA_002220535.1 | *Serratia marcescens* | UMH8 | Homo sapiens | University of Michigan | USA, Michigan | 2013 |
| GCA_002220555.1 | *Serratia marcescens* | UMH9 | Homo sapiens |  | USA, Michigan | 2014 |
| GCA_002220575.1 | *Serratia marcescens* | UMH11 | Homo sapiens | University of Michigan | USA, Michigan | 2014 |
| GCA_002220595.1 | *Serratia marcescens* | UMH12 | Homo sapiens | University of Michigan | USA, Michigan | 2014 |
| GCA_002220615.1 | *Serratia marcescens* | UMH1 | Homo sapiens |  | USA, Michigan | 2013 |
| GCA_002220635.1 | *Serratia marcescens* | UMH5 | Homo sapiens |  | USA, Michigan | 2014 |
| GCA_002220655.1 | *Serratia marcescens* | UMH3 | Homo sapiens | University of Michigan | USA, Michigan | 2014 |
| GCA_002220675.1 | *Serratia marcescens* | UMH6 | Homo sapiens | University of Michigan | USA, Michigan | 2013 |
| GCA_002220695.1 | *Serratia marcescens* | UMH10 | Homo sapiens | University of Michigan | USA, Michigan | 2014 |
| GCA_002220715.1 | *Serratia marcescens* | UMH7 | Homo sapiens |  | USA, Michigan | 2013 |
| GCA_002947235.1 | *Serratia marcescens* | AR_0027 |  |  |  |  |
| GCA_002996885.1 | *Serratia marcescens* | AR_0091 |  |  |  |  |
| GCA_002997125.1 | *Serratia marcescens* | AR_0099 |  |  |  |  |
| GCA_003031545.1 | *Serratia marcescens* | 95 | Homo sapiens | Sputum | USA, Boston | 2015 |
| GCA_003031645.1 | *Serratia marcescens* | BWH_35 | Homo sapiens | Sputum | USA, Boston | 2012 |
| GCA_003071565.1 | *Serratia marcescens* | AR_0124 |  |  |  |  |
| GCA_003071585.1 | *Serratia marcescens* | AR_0130 |  |  |  |  |
| GCA_003071605.1 | *Serratia marcescens* | AR_0123 |  |  |  |  |
| GCA_003071625.1 | *Serratia marcescens* | AR_0121 |  |  |  |  |
| GCA_003146705.1 | *Serratia marcescens* | CAV1761 | Homo sapiens | Peri rectal | USA, Virginia | 2014 |
| GCA_003182655.1 | *Serratia marcescens* | SGAir0764 |  | Air | Singapore | 2016 |
| GCA_003186475.1 | *Serratia marcescens* | 332 | Homo sapiens | Wound | USA, Boston | 2016 |
| GCA_003204075.1 | *Serratia marcescens* | AR_0131 |  |  |  |  |
| GCA_003204405.1 | *Serratia marcescens* | AR_0122 |  |  |  |  |
| GCA_003355135.1 | *Serratia marcescens* | N4_5 |  | Soil | USA, New Jersey | 1995 |
| GCA_003967055.1 | *Serratia marcescens* | AS_1 |  | Soil | Japan, Tochigi |  |
| GCA_006711125.1 | *Serratia marcescens* | WVU_004 | Homo sapiens | Blood | USA, Morgantown | 2019 |
| GCA_006711145.1 | *Serratia marcescens* | WVU_005 | Homo sapiens | Blood | USA, Morgantown | 2019 |
| GCA_006711245.1 | *Serratia marcescens* | WVU_006 | Homo sapiens | Blood | USA, Morgantown | 2019 |
| GCA_006711405.1 | *Serratia marcescens* | WVU_007 | Homo sapiens | Blood | USA, Morgantown | 2019 |
| GCA_006711525.1 | *Serratia marcescens* | WVU_008 | Homo sapiens | Blood | USA, Morgantown | 2019 |
| GCA_006716725.1 | *Serratia marcescens* | WVU_009 | Homo sapiens | Blood | USA, Morgantown | 2019 |
| GCA_006716825.1 | *Serratia marcescens* | WVU_010 | Homo sapiens | Blood | USA, Morgantown | 2019 |
| GCA_006842785.1 | *Serratia marcescens* | WVU_002 | Homo sapiens | Blood | USA, Morgantown | 2018 |
| GCA_008364265.2 | *Serratia marcescens* | S7_1 |  | Mixed salads | Germany | 2015 |
| GCA_008931425.1 | *Serratia marcescens* | E28 |  | Ensuite | Australia, Sydney | 2012 |
| GCA_009834305.1 | *Serratia marcescens* | N10A28 | Apis mellifera |  | USA, Connecticut | 2011 |
| GCA_009858195.1 | *Serratia marcescens* | 1602 | Homo sapiens |  | China, Zhengzhou | 2018 |
| GCA_009909345.1 | *Serratia marcescens* | 4201 | Homo sapiens | Sputum | China | 2019 |
| GCA_009909365.1 | *Serratia marcescens* | 3024 | Homo sapiens | Blood | China | 2018 |
| GCA_009909385.1 | *Serratia marcescens* | 1140_ | Homo sapiens | Body fluid | China | 2018 |
| GCA_009909405.1 | *Serratia marcescens* | 2838 | Homo sapiens | Body fluid | China | 2018 |
| GCA_009909425.1 | *Serratia marcescens* | C110 | Homo sapiens | Sputum | China | 2018 |
| GCA_009936295.1 | *Serratia marcescens* | ATCC_274 |  |  |  |  |
| GCA_011602465.1 | *Serratia marcescens* | BP2 | Jatropha curcas | Seeds | Brazil | 2010 |
| GCA_011769885.1 | *Serratia marcescens* | SER00094 | Homo sapiens | Sputum | USA, Pennsylvania,Pittsburgh | 2017 |
| GCA_013112395.1 | *Serratia marcescens* | FZSF02 |  | Soil | China, Fuzhou | 2014 |
| GCA_013122155.1 | *Serratia marcescens* | FY | Drosophila melanogaster |  | China, Shanxi | 2016 |
| GCA_013256815.1 | *Serratia marcescens* | LY1 | insect |  | China, muchuan | 2019 |
| GCA_013367735.1 | *Serratia marcescens* | JW_CZ2 |  | Rhizosphere soil | China, anhui | 2014 |
| GCA_013377375.1 | *Serratia marcescens* | 1912768R |  | Rhizosphere soil | China, Sichuan | 2015 |
| GCA_013426135.1 | *Serratia marcescens* | 12/2010 | Homo sapiens | Platelet concentrate | Canada, Ottawa | 2010 |
| GCA_013426155.1 | *Serratia marcescens* | 11/2010 | Homo sapiens | Platelet concentrate | Canada, Ottawa | 2010 |
| GCA_015074945.1 | *Serratia marcescens* | SCH909 | Homo sapiens |  | Greece | 1988 |
| GCA_015160915.1 | *Serratia marcescens* | SCQ1 | silkworm | Silkworm | China, Chongqing | 2009 |
| GCA_015708655.1 | *Serratia marcescens* | Byron | Curculio caryae | Pupal cells | USA, Georgia,Byron | 2014 |
| GCA_904866365.1 | *Serratia marcescens* | MSB1_9C |  |  |  |  |
| GCA_000828775.1 | *Serratia marcescens* | SM39 |  |  |  |  |
| GCA_900029885.1 | *Serratia marcescens* | SMB2099 |  | Clinical isolate |  |  |
| GCA_006974205.1 | *Serratia marcescens* | ATCC_13880^a^ |  | Pond water | USA | 1969 |
| GCA_000513215.1 | *Serratia marcescens* | Db11 |  |  |  |  |
| GCA_000336425.1 | *Serratia marcescens* | WW4 |  |  |  |  |
| GCA_004768745.1 | *Serratia nematodiphila* | DH_S01 |  |  |  |  |
| GCA_900635445.1 | *Serratia odorifera* | NCTC11214^a^ |  |  |  |  |
| GCA_001663115.1 | *Serratia plymuthica* | 3Rp8 | Brassica napus | Organic material | Germany, Braunschweig | 1998 |
| GCA_001663135.1 | *Serratia plymuthica* | 3Re4_18 | Solanum tuberosum | Organic material | Germany, Bonn | 2001 |
| GCA_013122215.1 | *Serratia plymuthica* | C_1 | Capsicum annuum | Red pepper | South Korea, Naju | 1998 |
| GCA_016027115.1 | *Serratia plymuthica* | FDAARGOS_907 |  |  |  |  |
| GCA_016027595.1 | *Serratia plymuthica* | FDAARGOS_896 |  |  |  |  |
| GCA_016027675.1 | *Serratia plymuthica* | FDAARGOS_895 |  |  |  |  |
| GCA_016027835.1 | *Serratia plymuthica* | FDAARGOS_889 |  |  |  |  |
| GCA_016726325.1 | *Serratia plymuthica* | FDAARGOS_1138 |  |  | Germany, Braunschweig |  |
| GCA_900478125.1 | *Serratia plymuthica* | NCTC12961^a^ |  |  |  |  |
| GCA_900635625.1 | *Serratia plymuthica* | NCTC8900 |  |  |  |  |
| GCA_900637965.1 | *Serratia plymuthica* | NCTC8015 |  | Canal water |  |  |
| GCA_000176835.2 | *Serratia plymuthica* | 4Rx13 |  |  |  |  |
| GCA_000214235.1 | *Serratia plymuthica* | AS9 |  |  |  |  |
| GCA_000261045.2 | *Serratia plymuthica* | PRI_2c |  | Rhizosphere soil | Netherlands | 2004 |
| GCA_000438825.1 | *Serratia plymuthica* | S13 |  | Styrian pumpkin |  |  |
| GCA_009660185.1 | *Serratia proteamaculans* | 336X |  | Leaf | China | 2017 |
| GCA_000018085.1 | *Serratia proteamaculans* | 568 |  |  |  |  |
| GCA_004684265.1 | *Serratia quinivorans* | PKL_12 |  | Rhizosphere soil | India, Lahaul Spiti | 2015 |
| GCA_900638135.1 | *Serratia quinivorans* | NCTC13188 |  |  |  |  |
| GCA_001572725.1 | *Serratia rubidaea* | 1122 | Homo sapiens | Sputum | China, Beijing | 2014 |
| GCA_016026735.1 | *Serratia rubidaea* | FDAARGOS_926 |  |  |  |  |
| GCA_016028475.1 | *Serratia rubidaea* | FDAARGOS_880 |  |  |  |  |
| GCA_900478395.1 | *Serratia rubidaea* | NCTC10848 |  |  |  |  |
| GCA_900635665.1 | *Serratia rubidaea* | NCTC9419 |  |  |  |  |
| GCA_900638005.1 | *Serratia rubidaea* | NCTC10036 |  | Finger |  |  |
| GCA_901472405.1 | *Serratia rubidaea* | NCTC12971^a^ |  |  |  |  |
| GCA_003641105.1 | *Serratia* sp. | 1D1416 | Euonymus japonicus | Gall tissue |  | 1972 |
| GCA_003668775.1 | *Serratia* sp. | 3ACOL1 | Cerambycidae sp. | Larvae | Norway | 2017 |
| GCA_000214195.1 | *Serratia* sp. | AS12 | rapeseed plant |  |  |  |
| GCA_000214805.1 | *Serratia* sp. | AS13 | rapeseed plant |  |  |  |
| GCA_003812745.1 | *Serratia* sp. | FDAARGOS_506 | Homo sapiens | Endotracheal aspirate |  | 2015 |
| GCA_000330865.1 | *Serratia* sp. | FGI94 |  |  |  |  |
| GCA_000695995.1 | *Serratia* sp. | FS14 |  |  |  |  |
| GCA_009817885.1 | *Serratia* sp. | KUDC3025 | Artemisia japonica | Rhizosphere soil | South Korea | 2017 |
| GCA_003719595.1 | *Serratia* sp. | LS_1 | Orthaga achatina |  |  | 2016 |
| GCA_002966855.1 | *Serratia* sp. | MYb239 |  | Compost | Germany, Kiel |  |
| GCA_009905295.1 | *Serratia* sp. | NGAS9 | Solanum tuberosum | Rhizosphere soil | Tanzania | 2018 |
| GCA_003691565.1 | *Serratia* sp. | P2ACOL2 | Cerambycidae sp. | Larvae | Norway | 2017 |
| GCA_000747565.1 | *Serratia* sp. | SCBI |  |  |  |  |
| GCA_002935055.1 | *Serratia* sp. | SSNIH1 |  |  | USA | 2015 |
| GCA_905188235.1 | *Serratia* sp. | Tan611 |  | Activated sludge |  |  |
| GCA_001642805.2 | *Serratia surfactantfaciens* | YD25^a^ |  | Rhizosphere soil | China, Fujian | 2011 |
| GCA_014304635.1 | *Serratia ureilytica* | CC119 | plant |  | USA, TX, Corpus Christi | 2017 |
| GCA_016728045.1 | *Serratia ureilytica* | FDAARGOS_1089 |  |  | Germany, Langen |  |

^a^ *Serratia* spp. type strains as listed in List of Prokaryotic names with Standing in Nomenclature (LPSN) [1]

1. Parte AC, Sardà Carbasse J, Meier-Kolthoff JP, Reimer LC, Göker M. List of Prokaryotic names with Standing in Nomenclature (LPSN) moves to the DSMZ. International Journal of Systematic and Evolutionary Microbiology. 2020;70(11):5607-12. doi: <https://doi.org/10.1099/ijsem.0.004332>.
