# Supplementary material for: Genomic characterisation of an entomopathogenic strain of Serratia ureilytica in the critically endangered phasmid Dryococelus australis
Source: PLoS One. 2022 Apr 20;17(4):e0265967. doi: 10.1371/journal.pone.0265967 (PMC9020675; doi:10.1371/journal.pone.0265967)
Supplement: S2 Table — (DOCX) [file pone.0265967.s006.docx]

**S2 Table. Features of the 708 partial or complete *Serratia* genomes used for phylogenomic and pan-genome analyses.**

| Genbank Assembly | Species | Strain | Host | Source | Country | Date |
| --- | --- | --- | --- | --- | --- | --- |
| GCA_003386805.1 | *S. ficaria* | WS3237 |  |  |  |  |
| GCA_900187015.1 | *S. ficaria* | NCTC12148 |  |  |  |  |
| GCA_001590885.1 | *S. ficaria* | NBRC_102596 |  |  |  |  |
| GCA_001006005.1 | *S. fonticola* | DSM_4576 |  | Water |  | 1979 |
| GCA_003353605.1 | *S. fonticola* | E2309 |  |  | Italy |  |
| GCA_006714955.1 | *S. fonticola* | 133R |  |  | USA |  |
| GCA_006715025.1 | *S. fonticola* | 119RB |  |  | USA |  |
| GCA_007827525.1 | *S. fonticola* | 128R |  |  | USA |  |
| GCA_900638145.1 | *S. fonticola* | NCTC13193 |  | Not available to be reported later |  |  |
| GCA_901482655.1 | *S. fonticola* | NCTC12965 |  | Spring water |  |  |
| GCA_000477615.1 | *S. fonticola* | AUAP2C |  |  |  |  |
| GCA_000468075.1 | *S. fonticola* | AUP3_3_ |  |  |  |  |
| GCA_000469035.1 | *S. fonticola* | LMG_7882 |  | Freshwater | Portugal |  |
| GCA_000476315.1 | *S. fonticola* | UTAD54 |  | Drinking water fountain | Portugal |  |
| GCA_005489985.1 | *S. fonticola* | MS5 | *Aedes aegypti* Rockefeller strain |  | USA | 2017 |
| GCA_001880985.1 | *S. fonticola* | AeS1 | *Aedes albopictus* |  | USA | 2015 |
| GCA_001908045.1 | *S. fonticola* | 5l | *Alces alces* |  | Russia | 2010 |
| GCA_002269665.1 | *S. fonticola* | BWK15 | *Anas penelope* | Feces | Japan | 2017 |
| GCA_001514455.1 | *S. fonticola* | GS2 | Sesame | Soil | South Korea | 2015 |
| GCA_002588845.1 | *S. fonticola* | FDAARGOS_411 | Wildlife | Liver tissue |  |  |
| GCA_000734885.1 | *S. grimesii* | A2 |  | Buffer solution | Russia | 1980 |
| GCA_003530495.1 | *S. grimesii* |  |  | Metagenome |  |  |
| GCA_900186025.1 | *S. grimesii* |  |  | Diseased Pinus pinaster tree in |  | 2009 |
| GCA_900456935.1 | *S. grimesii* | NCTC11543 |  |  |  |  |
| GCA_001590905.1 | *S. grimesii* | NBRC_13537 |  |  |  |  |
| GCA_003524615.1 | *S. liquefaciens* |  |  | Metagenome |  |  |
| GCA_004153815.1 | *S. liquefaciens* | B41552 |  | Ground beef | USA | 2016 |
| GCA_004153825.1 | *S. liquefaciens* | B41184 |  | Asparagus | USA | 2016 |
| GCA_004153865.1 | *S. liquefaciens* | B41553 |  | Ground beef | USA | 2016 |
| GCA_004153875.1 | *S. liquefaciens* | B41555 |  | Ground beef | USA | 2016 |
| GCA_004153885.1 | *S. liquefaciens* | B41623 |  | Raw shrimp | USA | 2016 |
| GCA_008364325.1 | *S. liquefaciens* | S1 |  | Mixed salads | Germany | 2015 |
| GCA_900706805.1 | *S. liquefaciens* | NCTC12962 |  | Not available not collected |  |  |
| GCA_000422085.1 | *S. liquefaciens* | ATCC_27592 |  |  |  |  |
| GCA_000699165.1 | *S. liquefaciens* | FK01 |  |  |  |  |
| GCA_003074975.1 | *S. liquefaciens* | JL02 | cattle | Milk from cattle farm | China | 2017 |
| GCA_003075015.1 | *S. liquefaciens* | JL03 | cow | Farm | China | 2017 |
| GCA_000975245.1 | *S. liquefaciens* | HUMV21 | Human | Skin ulcer | Spain | 2009 |
| GCA_001062925.1 | *S. liquefaciens* | 20_SPLY | Human |  | USA |  |
| GCA_001559135.2 | *S. liquefaciens* | FDAARGOS_125 | Human |  | USA | 2013 |
| GCA_001908115.1 | *S. liquefaciens* | 72 | *Pygoscelis adeliae* | Guano | Antarctica | 2011 |
| GCA_006970665.1 | *S. liquefaciens* | FG3 | *Stachytarpheta glabra* |  | Brazil | 2016 |
| GCA_000695485.1 | *S. marcescens* | ATCC_14041 |  |  | USA | 2013 |
| GCA_000743395.1 | *S. marcescens* | CDC_81360 |  |  |  |  |
| GCA_000751195.1 | *S. marcescens* |  |  |  |  |  |
| GCA_001007555.1 | *S. marcescens* | 90166 |  |  | USA | 1990 |
| GCA_001536325.1 | *S. marcescens* | 2880STDY5682955 |  | Blood | United Kingdom | 2008 |
| GCA_001536345.1 | *S. marcescens* | 2880STDY5682861 |  | Blood | United Kingdom | 2003 |
| GCA_001536365.1 | *S. marcescens* | 2880STDY5682980 |  | Blood | United Kingdom | 2009 |
| GCA_001536385.1 | *S. marcescens* | 2880STDY5682927 |  | Blood | United Kingdom | 2006 |
| GCA_001536405.1 | *S. marcescens* | 2880STDY5682928 |  | Blood | United Kingdom | 2006 |
| GCA_001536425.1 | *S. marcescens* | 2880STDY5682998 |  | Blood | United Kingdom | 2010 |
| GCA_001536445.1 | *S. marcescens* | 2880STDY5682856 |  | Blood | United Kingdom | 2003 |
| GCA_001536465.1 | *S. marcescens* | 2880STDY5682864 |  | Blood | United Kingdom | 2004 |
| GCA_001536485.1 | *S. marcescens* | 2880STDY5682815 |  | Blood | United Kingdom | 2001 |
| GCA_001536505.1 | *S. marcescens* | 2880STDY5682857 |  | Blood | United Kingdom | 2003 |
| GCA_001536525.1 | *S. marcescens* | 2880STDY5682952 |  | Blood | United Kingdom | 2008 |
| GCA_001536545.1 | *S. marcescens* | 2880STDY5682994 |  | Blood | United Kingdom | 2010 |
| GCA_001536565.1 | *S. marcescens* | 2880STDY5682999 |  | Blood | United Kingdom | 2010 |
| GCA_001536585.1 | *S. marcescens* | 2880STDY5682922 |  | Blood | United Kingdom | 2006 |
| GCA_001536605.1 | *S. marcescens* | 2880STDY5683026 |  | Blood | United Kingdom | 2008 |
| GCA_001536625.1 | *S. marcescens* | 2880STDY5682959 |  | Blood | United Kingdom | 2008 |
| GCA_001536645.1 | *S. marcescens* | 2880STDY5682977 |  | Blood | United Kingdom | 2009 |
| GCA_001536665.1 | *S. marcescens* | 2880STDY5682960 |  | Blood | United Kingdom | 2008 |
| GCA_001536685.1 | *S. marcescens* | 2880STDY5682882 |  | Blood | United Kingdom | 2004 |
| GCA_001536705.1 | *S. marcescens* | 2880STDY5682964 |  | Blood | United Kingdom | 2008 |
| GCA_001536725.1 | *S. marcescens* | 2880STDY5682906 |  | Blood | United Kingdom | 2005 |
| GCA_001536745.1 | *S. marcescens* | 2880STDY5682992 |  | Blood | United Kingdom | 2010 |
| GCA_001536765.1 | *S. marcescens* | 2880STDY5682859 |  | Blood | United Kingdom | 2003 |
| GCA_001536845.1 | *S. marcescens* | 2880STDY5682929 |  | Blood | United Kingdom | 2006 |
| GCA_001536895.1 | *S. marcescens* | 2880STDY5682950 |  | Blood | United Kingdom | 2007 |
| GCA_001536945.1 | *S. marcescens* | 2880STDY5682966 |  | Blood | United Kingdom | 2008 |
| GCA_001536965.1 | *S. marcescens* | 2880STDY5682942 |  | Blood | United Kingdom | 2007 |
| GCA_001536985.1 | *S. marcescens* | 2880STDY5682874 |  | Blood | United Kingdom | 2004 |
| GCA_001537005.1 | *S. marcescens* | 2880STDY5682951 |  | Blood | United Kingdom | 2007 |
| GCA_001537025.1 | *S. marcescens* | 2880STDY5682846 |  | Blood | United Kingdom | 2003 |
| GCA_001537045.1 | *S. marcescens* | 2880STDY5682914 |  | Blood | United Kingdom | 2006 |
| GCA_001537065.1 | *S. marcescens* | 2880STDY5682905 |  | Blood | United Kingdom | 2005 |
| GCA_001537085.1 | *S. marcescens* | 2880STDY5683001 |  | Blood | United Kingdom | 2010 |
| GCA_001537105.1 | *S. marcescens* | 2880STDY5683024 |  | Blood | United Kingdom | 2011 |
| GCA_001537125.1 | *S. marcescens* | 2880STDY5682916 |  | Blood | United Kingdom | 2006 |
| GCA_001537145.1 | *S. marcescens* | 2880STDY5682819 |  | Blood | United Kingdom | 2002 |
| GCA_001537165.1 | *S. marcescens* | 2880STDY5682944 |  | Blood | United Kingdom | 2007 |
| GCA_001537185.1 | *S. marcescens* | 2880STDY5682924 |  | Blood | United Kingdom | 2006 |
| GCA_001537205.1 | *S. marcescens* | 2880STDY5682828 |  | Blood | United Kingdom | 2002 |
| GCA_001537225.1 | *S. marcescens* | 2880STDY5682866 |  | Blood | United Kingdom | 2004 |
| GCA_001537245.1 | *S. marcescens* | 2880STDY5682871 |  | Blood | United Kingdom | 2004 |
| GCA_001537285.1 | *S. marcescens* | 2880STDY5683022 |  | Blood | United Kingdom | 2011 |
| GCA_001537305.1 | *S. marcescens* | 2880STDY5682930 |  | Blood | United Kingdom | 2007 |
| GCA_001537325.1 | *S. marcescens* | 2880STDY5682845 |  | Blood | United Kingdom | 2003 |
| GCA_001537345.1 | *S. marcescens* | 2880STDY5682872 |  | Blood | United Kingdom | 2004 |
| GCA_001537365.1 | *S. marcescens* | 2880STDY5682897 |  | Blood | United Kingdom | 2005 |
| GCA_001537405.1 | *S. marcescens* | 2880STDY5683035 |  | Blood | United Kingdom | 2006 |
| GCA_001537425.1 | *S. marcescens* | 2880STDY5682840 |  | Blood | United Kingdom | 2003 |
| GCA_001537445.1 | *S. marcescens* | 2880STDY5683014 |  | Blood | United Kingdom | 2010 |
| GCA_001537465.1 | *S. marcescens* | 2880STDY5682878 |  | Blood | United Kingdom | 2004 |
| GCA_001537485.1 | *S. marcescens* | 2880STDY5682869 |  | Blood | United Kingdom | 2004 |
| GCA_001537505.1 | *S. marcescens* | 2880STDY5682898 |  | Blood | United Kingdom | 2005 |
| GCA_001537525.1 | *S. marcescens* | 2880STDY5682816 |  | Blood | United Kingdom | 2001 |
| GCA_001537545.1 | *S. marcescens* | 2880STDY5682911 |  | Blood | United Kingdom | 2006 |
| GCA_001537565.1 | *S. marcescens* | 2880STDY5683034 |  | Blood | United Kingdom | 2006 |
| GCA_001537585.1 | *S. marcescens* | 2880STDY5682974 |  | Blood | United Kingdom | 2009 |
| GCA_001537605.1 | *S. marcescens* | 2880STDY5682851 |  | Blood | United Kingdom | 2003 |
| GCA_001537625.1 | *S. marcescens* | 2880STDY5682926 |  | Blood | United Kingdom | 2006 |
| GCA_001537645.1 | *S. marcescens* | 2880STDY5682830 |  | Blood | United Kingdom | 2002 |
| GCA_001537665.1 | *S. marcescens* | 2880STDY5682881 |  | Blood | United Kingdom | 2004 |
| GCA_001537685.1 | *S. marcescens* | 2880STDY5682853 |  | Blood | United Kingdom | 2003 |
| GCA_001537705.1 | *S. marcescens* | 2880STDY5682986 |  | Blood | United Kingdom | 2009 |
| GCA_001537725.1 | *S. marcescens* | 2880STDY5682900 |  | Blood | United Kingdom | 2005 |
| GCA_001537745.1 | *S. marcescens* | 2880STDY5683003 |  | Blood | United Kingdom | 2010 |
| GCA_001537765.1 | *S. marcescens* | 2880STDY5682820 |  | Blood | United Kingdom | 2002 |
| GCA_001537785.1 | *S. marcescens* | 2880STDY5683006 |  | Blood | United Kingdom | 2010 |
| GCA_001537805.1 | *S. marcescens* | 2880STDY5683016 |  | Blood | United Kingdom | 2010 |
| GCA_001537825.1 | *S. marcescens* | 2880STDY5683030 |  | Blood | United Kingdom | 2003 |
| GCA_001537845.1 | *S. marcescens* | 2880STDY5682823 |  | Blood | United Kingdom | 2002 |
| GCA_001537865.1 | *S. marcescens* | 2880STDY5682949 |  | Blood | United Kingdom | 2007 |
| GCA_001537885.1 | *S. marcescens* | 2880STDY5682825 |  | Blood | United Kingdom | 2002 |
| GCA_001537905.1 | *S. marcescens* | 2880STDY5682876 |  | Blood | United Kingdom | 2004 |
| GCA_001537925.1 | *S. marcescens* | 2880STDY5682995 |  | Blood | United Kingdom | 2010 |
| GCA_001538005.1 | *S. marcescens* | 2880STDY5682947 |  | Blood | United Kingdom | 2007 |
| GCA_001538115.1 | *S. marcescens* | 2880STDY5682971 |  | Blood | United Kingdom | 2009 |
| GCA_001538195.1 | *S. marcescens* | 2880STDY5683020 |  | Blood | United Kingdom | 2011 |
| GCA_001538275.1 | *S. marcescens* | 2880STDY5682912 |  | Blood | United Kingdom | 2006 |
| GCA_001538325.1 | *S. marcescens* | 2880STDY5682939 |  | Blood | United Kingdom | 2007 |
| GCA_001538345.1 | *S. marcescens* | 2880STDY5682888 |  | Blood | United Kingdom | 2005 |
| GCA_001538365.1 | *S. marcescens* | 2880STDY5682887 |  | Blood | United Kingdom | 2005 |
| GCA_001538385.1 | *S. marcescens* | 2880STDY5682835 |  | Blood | United Kingdom | 2002 |
| GCA_001538405.1 | *S. marcescens* | 2880STDY5682968 |  | Blood | United Kingdom | 2009 |
| GCA_001538425.1 | *S. marcescens* | 2880STDY5682868 |  | Blood | United Kingdom | 2004 |
| GCA_001538445.1 | *S. marcescens* | 2880STDY5682848 |  | Blood | United Kingdom | 2003 |
| GCA_001538465.1 | *S. marcescens* | 2880STDY5683010 |  | Blood | United Kingdom | 2010 |
| GCA_001538485.1 | *S. marcescens* | 2880STDY5683021 |  | Blood | United Kingdom | 2011 |
| GCA_001538505.1 | *S. marcescens* | 2880STDY5682945 |  | Blood | United Kingdom | 2007 |
| GCA_001538525.1 | *S. marcescens* | 2880STDY5682824 |  | Blood | United Kingdom | 2002 |
| GCA_001538545.1 | *S. marcescens* | 2880STDY5683017 |  | Blood | United Kingdom | 2010 |
| GCA_001538565.1 | *S. marcescens* | 2880STDY5683004 |  | Blood | United Kingdom | 2010 |
| GCA_001538585.1 | *S. marcescens* | 2880STDY5682979 |  | Blood | United Kingdom | 2009 |
| GCA_001538605.1 | *S. marcescens* | 2880STDY5682822 |  | Blood | United Kingdom | 2002 |
| GCA_001538625.1 | *S. marcescens* | 2880STDY5682836 |  | Blood | United Kingdom | 2002 |
| GCA_001538645.1 | *S. marcescens* | 2880STDY5682907 |  | Blood | United Kingdom | 2006 |
| GCA_001538665.1 | *S. marcescens* | 2880STDY5682975 |  | Blood | United Kingdom | 2009 |
| GCA_001538685.1 | *S. marcescens* | 2880STDY5682967 |  | Blood | United Kingdom | 2008 |
| GCA_001538705.1 | *S. marcescens* | 2880STDY5683032 |  | Blood | United Kingdom | 2006 |
| GCA_001538725.1 | *S. marcescens* | 2880STDY5682852 |  | Blood | United Kingdom | 2003 |
| GCA_001538745.1 | *S. marcescens* | 2880STDY5682934 |  | Blood | United Kingdom | 2007 |
| GCA_001538765.1 | *S. marcescens* | 2880STDY5682826 |  | Blood | United Kingdom | 2002 |
| GCA_001538785.1 | *S. marcescens* | 2880STDY5683025 |  | Blood | United Kingdom | 2011 |
| GCA_001538805.1 | *S. marcescens* | 2880STDY5682847 |  | Blood | United Kingdom | 2003 |
| GCA_001538825.1 | *S. marcescens* | 2880STDY5682883 |  | Blood | United Kingdom | 2004 |
| GCA_001538845.1 | *S. marcescens* | 2880STDY5682996 |  | Blood | United Kingdom | 2010 |
| GCA_001538865.1 | *S. marcescens* | 2880STDY5682993 |  | Blood | United Kingdom | 2010 |
| GCA_001538885.1 | *S. marcescens* | 2880STDY5682931 |  | Blood | United Kingdom | 2007 |
| GCA_001538905.1 | *S. marcescens* | 2880STDY5682814 |  | Blood | United Kingdom | 2001 |
| GCA_001538925.1 | *S. marcescens* | 2880STDY5682901 |  | Blood | United Kingdom | 2005 |
| GCA_001538945.1 | *S. marcescens* | 2880STDY5682933 |  | Blood | United Kingdom | 2007 |
| GCA_001538965.1 | *S. marcescens* | 2880STDY5682938 |  | Blood | United Kingdom | 2007 |
| GCA_001538985.1 | *S. marcescens* | 2880STDY5683036 |  | Blood | United Kingdom | 2006 |
| GCA_001539005.1 | *S. marcescens* | 2880STDY5682873 |  | Blood | United Kingdom | 2004 |
| GCA_001539025.1 | *S. marcescens* | 2880STDY5682818 |  | Blood | United Kingdom | 2002 |
| GCA_001539045.1 | *S. marcescens* | 2880STDY5682935 |  | Blood | United Kingdom | 2007 |
| GCA_001539065.1 | *S. marcescens* | 2880STDY5682988 |  | Blood | United Kingdom | 2010 |
| GCA_001539085.1 | *S. marcescens* | 2880STDY5682904 |  | Blood | United Kingdom | 2005 |
| GCA_001539105.1 | *S. marcescens* | 2880STDY5683015 |  | Blood | United Kingdom | 2010 |
| GCA_001539125.1 | *S. marcescens* | 2880STDY5683011 |  | Blood | United Kingdom | 2010 |
| GCA_001539145.1 | *S. marcescens* | 2880STDY5682908 |  | Blood | United Kingdom | 2006 |
| GCA_001539185.1 | *S. marcescens* | 2880STDY5683009 |  | Blood | United Kingdom | 2010 |
| GCA_001539205.1 | *S. marcescens* | 2880STDY5682962 |  | Blood | United Kingdom | 2008 |
| GCA_001539225.1 | *S. marcescens* | 2880STDY5683013 |  | Blood | United Kingdom | 2010 |
| GCA_001539245.1 | *S. marcescens* | 2880STDY5682921 |  | Blood | United Kingdom | 2006 |
| GCA_001539265.1 | *S. marcescens* | 2880STDY5682854 |  | Blood | United Kingdom | 2003 |
| GCA_001539285.1 | *S. marcescens* | 2880STDY5682936 |  | Blood | United Kingdom | 2007 |
| GCA_001539305.1 | *S. marcescens* | 2880STDY5683007 |  | Blood | United Kingdom | 2010 |
| GCA_001539325.1 | *S. marcescens* | 2880STDY5682956 |  | Blood | United Kingdom | 2008 |
| GCA_001539345.1 | *S. marcescens* | 2880STDY5682884 |  | Blood | United Kingdom | 2004 |
| GCA_001539365.1 | *S. marcescens* | 2880STDY5682850 |  | Blood | United Kingdom | 2003 |
| GCA_001539385.1 | *S. marcescens* | 2880STDY5682913 |  | Blood | United Kingdom | 2006 |
| GCA_001539405.1 | *S. marcescens* | 2880STDY5682937 |  | Blood | United Kingdom | 2007 |
| GCA_001539425.1 | *S. marcescens* | 2880STDY5682877 |  | Blood | United Kingdom | 2004 |
| GCA_001539445.1 | *S. marcescens* | 2880STDY5682965 |  | Blood | United Kingdom | 2008 |
| GCA_001539465.1 | *S. marcescens* | 2880STDY5683000 |  | Blood | United Kingdom | 2010 |
| GCA_001539485.1 | *S. marcescens* | 2880STDY5683008 |  | Blood | United Kingdom | 2010 |
| GCA_001539505.1 | *S. marcescens* | 2880STDY5682831 |  | Blood | United Kingdom | 2002 |
| GCA_001539525.1 | *S. marcescens* | 2880STDY5683027 |  | Blood | United Kingdom | 2009 |
| GCA_001539545.1 | *S. marcescens* | 2880STDY5682953 |  | Blood | United Kingdom | 2008 |
| GCA_001539565.1 | *S. marcescens* | 2880STDY5682893 |  | Blood | United Kingdom | 2005 |
| GCA_001539585.1 | *S. marcescens* | 2880STDY5682863 |  | Blood | United Kingdom | 2004 |
| GCA_001539605.1 | *S. marcescens* | 2880STDY5682984 |  | Blood | United Kingdom | 2009 |
| GCA_001539625.1 | *S. marcescens* | 2880STDY5682943 |  | Blood | United Kingdom | 2007 |
| GCA_001539645.1 | *S. marcescens* | 2880STDY5682838 |  | Blood | United Kingdom | 2003 |
| GCA_001539665.1 | *S. marcescens* | 2880STDY5682954 |  | Blood | United Kingdom | 2008 |
| GCA_001539685.1 | *S. marcescens* | 2880STDY5682983 |  | Blood | United Kingdom | 2009 |
| GCA_001539705.1 | *S. marcescens* | 2880STDY5682843 |  | Blood | United Kingdom | 2003 |
| GCA_001539725.1 | *S. marcescens* | 2880STDY5682839 |  | Blood | United Kingdom | 2003 |
| GCA_001539745.1 | *S. marcescens* | 2880STDY5682889 |  | Blood | United Kingdom | 2005 |
| GCA_001539765.1 | *S. marcescens* | 2880STDY5682858 |  | Blood | United Kingdom | 2003 |
| GCA_001539785.1 | *S. marcescens* | 2880STDY5682827 |  | Blood | United Kingdom | 2002 |
| GCA_001539805.1 | *S. marcescens* | 2880STDY5682834 |  | Blood | United Kingdom | 2002 |
| GCA_001539825.1 | *S. marcescens* | 2880STDY5682978 |  | Blood | United Kingdom | 2009 |
| GCA_001539845.1 | *S. marcescens* | 2880STDY5682867 |  | Blood | United Kingdom | 2004 |
| GCA_001539865.1 | *S. marcescens* | 2880STDY5682903 |  | Blood | United Kingdom | 2005 |
| GCA_001539885.1 | *S. marcescens* | 2880STDY5682865 |  | Blood | United Kingdom | 2004 |
| GCA_001539905.1 | *S. marcescens* | 2880STDY5682961 |  | Blood | United Kingdom | 2008 |
| GCA_001539925.1 | *S. marcescens* | 2880STDY5682875 |  | Blood | United Kingdom | 2004 |
| GCA_001539945.1 | *S. marcescens* | 2880STDY5682963 |  | Blood | United Kingdom | 2008 |
| GCA_001539965.1 | *S. marcescens* | 2880STDY5682829 |  | Blood | United Kingdom | 2002 |
| GCA_001539985.1 | *S. marcescens* | 2880STDY5683033 |  | Blood | United Kingdom | 2006 |
| GCA_001540005.1 | *S. marcescens* | 2880STDY5682958 |  | Blood | United Kingdom | 2008 |
| GCA_001540025.1 | *S. marcescens* | 2880STDY5682940 |  | Blood | United Kingdom | 2007 |
| GCA_001540045.1 | *S. marcescens* | 2880STDY5682981 |  | Blood | United Kingdom | 2009 |
| GCA_001540065.1 | *S. marcescens* | 2880STDY5682990 |  | Blood | United Kingdom | 2010 |
| GCA_001540085.1 | *S. marcescens* | 2880STDY5682925 |  | Blood | United Kingdom | 2006 |
| GCA_001540105.1 | *S. marcescens* | 2880STDY5682902 |  | Blood | United Kingdom | 2005 |
| GCA_001540125.1 | *S. marcescens* | 2880STDY5683037 |  | Blood | United Kingdom | 2007 |
| GCA_001540165.1 | *S. marcescens* | 2880STDY5682997 |  | Blood | United Kingdom | 2010 |
| GCA_001540185.1 | *S. marcescens* | 2880STDY5682832 |  | Blood | United Kingdom | 2002 |
| GCA_001540205.1 | *S. marcescens* | 2880STDY5682842 |  | Blood | United Kingdom | 2003 |
| GCA_001540225.1 | *S. marcescens* | 2880STDY5683031 |  | Blood | United Kingdom | 2006 |
| GCA_001540245.1 | *S. marcescens* | 2880STDY5682918 |  | Blood | United Kingdom | 2006 |
| GCA_001540265.1 | *S. marcescens* | 2880STDY5682849 |  | Blood | United Kingdom | 2003 |
| GCA_001540285.1 | *S. marcescens* | 2880STDY5682917 |  | Blood | United Kingdom | 2006 |
| GCA_001540305.1 | *S. marcescens* | 2880STDY5682890 |  | Blood | United Kingdom | 2005 |
| GCA_001540325.1 | *S. marcescens* | 2880STDY5682891 |  | Blood | United Kingdom | 2005 |
| GCA_001540345.1 | *S. marcescens* | 2880STDY5682970 |  | Blood | United Kingdom | 2009 |
| GCA_001540365.1 | *S. marcescens* | 2880STDY5682862 |  | Blood | United Kingdom | 2003 |
| GCA_001540385.1 | *S. marcescens* | 2880STDY5682880 |  | Blood | United Kingdom | 2004 |
| GCA_001540405.1 | *S. marcescens* | 2880STDY5682948 |  | Blood | United Kingdom | 2007 |
| GCA_001540425.1 | *S. marcescens* | 2880STDY5682855 |  | Blood | United Kingdom | 2003 |
| GCA_001540445.1 | *S. marcescens* | 2880STDY5682976 |  | Blood | United Kingdom | 2009 |
| GCA_001540465.1 | *S. marcescens* | 2880STDY5682915 |  | Blood | United Kingdom | 2006 |
| GCA_001540485.1 | *S. marcescens* | 2880STDY5682919 |  | Blood | United Kingdom | 2006 |
| GCA_001540505.1 | *S. marcescens* | 2880STDY5682982 |  | Blood | United Kingdom | 2009 |
| GCA_001540525.1 | *S. marcescens* | 2880STDY5682870 |  | Blood | United Kingdom | 2004 |
| GCA_001540545.1 | *S. marcescens* | 2880STDY5682969 |  | Blood | United Kingdom | 2009 |
| GCA_001540565.1 | *S. marcescens* | 2880STDY5683002 |  | Blood | United Kingdom | 2010 |
| GCA_001540585.1 | *S. marcescens* | 2880STDY5682991 |  | Blood | United Kingdom | 2010 |
| GCA_001540605.1 | *S. marcescens* | 2880STDY5682879 |  | Blood | United Kingdom | 2004 |
| GCA_001540625.1 | *S. marcescens* | 2880STDY5682885 |  | Blood | United Kingdom | 2004 |
| GCA_001540665.1 | *S. marcescens* | 2880STDY5683018 |  | Blood | United Kingdom | 2010 |
| GCA_001540685.1 | *S. marcescens* | 2880STDY5682899 |  | Blood | United Kingdom | 2005 |
| GCA_001540705.1 | *S. marcescens* | 2880STDY5682973 |  | Blood | United Kingdom | 2009 |
| GCA_001540725.1 | *S. marcescens* | 2880STDY5682844 |  | Blood | United Kingdom | 2003 |
| GCA_001540745.1 | *S. marcescens* | 2880STDY5682896 |  | Blood | United Kingdom | 2005 |
| GCA_001540765.1 | *S. marcescens* | 2880STDY5682892 |  | Blood | United Kingdom | 2005 |
| GCA_001540785.1 | *S. marcescens* | 2880STDY5682987 |  | Blood | United Kingdom | 2009 |
| GCA_001540805.1 | *S. marcescens* | 2880STDY5682837 |  | Blood | United Kingdom | 2003 |
| GCA_001540825.1 | *S. marcescens* | 2880STDY5682957 |  | Blood | United Kingdom | 2008 |
| GCA_001564475.1 | *S. marcescens* | 2880STDY5682985 |  | Blood | United Kingdom | 2009 |
| GCA_001716895.1 | *S. marcescens* | UENF22GI |  | Vermicompost | Brazil | 2013 |
| GCA_001902635.1 | *S. marcescens* | MSU97 |  | Plant surface | Venezuela | 2016 |
| GCA_001940505.1 | *S. marcescens* | BJL200 |  |  |  |  |
| GCA_002029205.1 | *S. marcescens* | CAPREX_SY13 |  | Ghanaian yam | United Kingdom | 2016 |
| GCA_002029225.1 | *S. marcescens* | CAPREX_SY21 |  | Ghanaian yam | United Kingdom | 2016 |
| GCA_002094145.1 | *S. marcescens* | MEW06 |  | Lake water | China | 2014 |
| GCA_002108655.1 | *S. marcescens* | Z6 |  | Contaminated soil | China | 2016 |
| GCA_002205475.1 | *S. marcescens* | S2I7 |  | Soil | India | 2015 |
| GCA_002265665.1 | *S. marcescens* | EGDHP20_1 |  | Tannery waste | India | 2013 |
| GCA_002325865.1 | *S. marcescens* |  |  | Metal | USA |  |
| GCA_002872515.1 | *S. marcescens* | SOLR4 |  | Solanacea rhizosphere | Brazil | 2015 |
| GCA_002899335.1 | *S. marcescens* | JES_110 |  | Growing on Rhizopus stolonifer ioslated | USA | 2016 |
| GCA_002899355.1 | *S. marcescens* | ADJS2D_White | *Neoscapteriscus borellii* | Dead mole cricket | USA | 2015 |
| GCA_002899375.1 | *S. marcescens* | ADJS2C_Purple | *Neoscapteriscus borellii* | Dead mole cricket | USA | 2015 |
| GCA_002899415.1 | *S. marcescens* | ADJS2C_Red | *Neoscapteriscus borellii* | Dead mole cricket | USA | 2015 |
| GCA_002920375.1 | *S. marcescens* | ID148696 |  | Drain in NICU room | Canada |  |
| GCA_002920385.1 | *S. marcescens* | ID148299 |  | Around bed in NICU room | Canada |  |
| GCA_002947235.1 | *S. marcescens* | AR_0027 |  |  |  |  |
| GCA_002996885.1 | *S. marcescens* | AR_0091 |  |  |  |  |
| GCA_002997125.1 | *S. marcescens* | AR_0099 |  |  |  |  |
| GCA_003071565.1 | *S. marcescens* | AR_0124 |  |  |  |  |
| GCA_003071585.1 | *S. marcescens* | AR_0130 |  |  |  |  |
| GCA_003071605.1 | *S. marcescens* | AR_0123 |  |  |  |  |
| GCA_003071625.1 | *S. marcescens* | AR_0121 |  |  |  |  |
| GCA_003182655.1 | *S. marcescens* | SGAir0764 |  | Air | Singapore | 2016 |
| GCA_003204075.1 | *S. marcescens* | AR_0131 |  |  |  |  |
| GCA_003204405.1 | *S. marcescens* | AR_0122 |  |  |  |  |
| GCA_003241435.1 | *S. marcescens* |  |  | Hospital NICU surfaces and sink | USA | 2013 |
| GCA_003355135.1 | *S. marcescens* | N45 |  | Soil | USA | 1995 |
| GCA_003484665.1 | *S. marcescens* |  |  | Metagenome |  |  |
| GCA_003516165.1 | *S. marcescens* | KS10 |  | Marine | USA | 2006 |
| GCA_003516185.1 | *S. marcescens* | EL1 |  | Marine | USA | 2002 |
| GCA_003935125.1 | *S. marcescens* | SM_196 |  | Washroom sink in hospital intensive | Pakistan | 2016 |
| GCA_003935135.1 | *S. marcescens* | SM_321 |  | Nursing call button in hospital | Pakistan | 2016 |
| GCA_003935145.1 | *S. marcescens* | SM_362 |  | Nursing call button in hospital | Pakistan | 2016 |
| GCA_003935225.1 | *S. marcescens* | SM_153 |  | Alcohol foam dispenser in hospital | Pakistan | 2016 |
| GCA_003935235.1 | *S. marcescens* | SM_079 |  | Bedside light switch in hospital | Pakistan | 2016 |
| GCA_003935245.1 | *S. marcescens* | SM_076 |  | Nursing call button in hospital | Pakistan | 2016 |
| GCA_003935265.1 | *S. marcescens* | SM_188 |  | Washroom sink in hospital intensive | Pakistan | 2016 |
| GCA_003935355.1 | *S. marcescens* | SM_045 |  | Bedside light switch in hospital | Pakistan | 2016 |
| GCA_003935635.1 | *S. marcescens* | SM_037 |  | Nursing call button in hospital | Pakistan | 2016 |
| GCA_003935755.1 | *S. marcescens* | SM_204 |  | Bedside rail in hospital intensive care | Pakistan | 2016 |
| GCA_003935775.1 | *S. marcescens* | SM_190 |  | Alcohol foam dispenser in hospital | Pakistan | 2016 |
| GCA_003935805.1 | *S. marcescens* | SM_164 |  | Washroom sink in hospital intensive | Pakistan | 2016 |
| GCA_003935875.1 | *S. marcescens* | SM_090 |  | Nursing call button in hospital | Pakistan | 2016 |
| GCA_003957865.1 | *S. marcescens* | WWI31 |  | Marine | USA | 2006 |
| GCA_003957915.1 | *S. marcescens* | PDL100 |  | Marine | USA | 1999 |
| GCA_003957935.1 | *S. marcescens* | KS65 |  | Marine | USA | 2006 |
| GCA_003957945.1 | *S. marcescens* | KS40 |  | Marine | USA | 2006 |
| GCA_003957975.1 | *S. marcescens* | KS25 |  | Marine | USA | 2006 |
| GCA_003957985.1 | *S. marcescens* | KS23 |  | Marine | USA | 2006 |
| GCA_003958045.1 | *S. marcescens* | KS12 |  | Marine | USA | 2006 |
| GCA_003958065.1 | *S. marcescens* | KS9 |  | Marine | USA | 2006 |
| GCA_003958075.1 | *S. marcescens* | EL121 |  | Marine | USA | 2003 |
| GCA_003958085.1 | *S. marcescens* | KS1 |  | Marine | USA | 2006 |
| GCA_003958095.1 | *S. marcescens* | EL120 |  | Marine | USA | 2003 |
| GCA_003958135.1 | *S. marcescens* | EL118 |  | Marine | USA | 2003 |
| GCA_003958165.1 | *S. marcescens* | EL117 |  | Marine | USA | 2003 |
| GCA_003958175.1 | *S. marcescens* | EL115 |  | Marine | USA | 2003 |
| GCA_003958185.1 | *S. marcescens* | EL114 |  | Marine | USA | 2003 |
| GCA_003958205.1 | *S. marcescens* | EL113 |  | Marine | USA | 2003 |
| GCA_003958215.1 | *S. marcescens* | EL108 |  | Marine | USA | 2003 |
| GCA_003958265.1 | *S. marcescens* | EL98 |  | Marine | USA | 2003 |
| GCA_003958275.1 | *S. marcescens* | EL97 |  | Marine | USA | 2003 |
| GCA_003958295.1 | *S. marcescens* | EL96 |  | Marine | USA | 2003 |
| GCA_003958305.1 | *S. marcescens* | EL95 |  | Marine | USA | 2003 |
| GCA_003958335.1 | *S. marcescens* | EL85 |  | Marine | USA | 2003 |
| GCA_003958365.1 | *S. marcescens* | EL84 |  | Marine | USA | 2003 |
| GCA_003958385.1 | *S. marcescens* | EL3 |  | Marine | USA | 2002 |
| GCA_003958395.1 | *S. marcescens* | KS45 |  | Marine | USA | 2006 |
| GCA_003958405.1 | *S. marcescens* | KS16 |  | Marine | USA | 2006 |
| GCA_003958415.1 | *S. marcescens* | KS5 |  | Marine | USA | 2006 |
| GCA_003958445.1 | *S. marcescens* | EL122 |  | Marine | USA | 2003 |
| GCA_003958485.1 | *S. marcescens* | EL110 |  | Marine | USA | 2003 |
| GCA_003958495.1 | *S. marcescens* | EL109 |  | Marine | USA | 2003 |
| GCA_003958505.1 | *S. marcescens* | EL41 |  | Marine | USA | 2003 |
| GCA_003958535.1 | *S. marcescens* | EL6 |  | Marine | USA | 2002 |
| GCA_003958565.1 | *S. marcescens* | EL60 |  | Marine | USA | 2003 |
| GCA_003958575.1 | *S. marcescens* | EL116 |  | Marine | USA | 2003 |
| GCA_003958605.1 | *S. marcescens* | EL119 |  | Marine | USA | 2003 |
| GCA_003967055.1 | *S. marcescens* | AS1_Tochigi |  | Soil | Japan |  |
| GCA_004326135.1 | *S. marcescens* | SM6 |  | Soil |  | 1966 |
| GCA_006715005.1 | *S. marcescens* | 106R |  |  | USA |  |
| GCA_008364225.1 | *S. marcescens* | S13 |  | Lemon balm | Germany | 2015 |
| GCA_008364235.1 | *S. marcescens* | S18 |  | Cucumber | Germany | 2015 |
| GCA_008364245.1 | *S. marcescens* | S15 |  | Cucumber | Germany | 2015 |
| GCA_008364255.1 | *S. marcescens* | S10 |  | Basil | Germany | 2015 |
| GCA_008364265.1 | *S. marcescens* | S7.1 |  | Mixed salads | Germany | 2015 |
| GCA_008364335.1 | *S. marcescens* | S2 |  | Cucumber | Germany | 2015 |
| GCA_008931425.1 | *S. marcescens* | E28 |  | Ensuite 7 8 | Australia | 2012 |
| GCA_900108825.1 | *S. marcescens* |  |  |  |  |  |
| GCA_900108835.1 | *S. marcescens* |  |  |  |  |  |
| GCA_900456855.1 | *S. marcescens* | NCTC13920 |  |  |  |  |
| GCA_900456915.1 | *S. marcescens* | NCTC13382 |  |  |  |  |
| GCA_900457055.1 | *S. marcescens* | NCTC10211 ^a^ |  |  |  |  |
| GCA_900518895.1 | *S. marcescens* |  |  |  |  |  |
| GCA_900518925.1 | *S. marcescens* |  |  |  |  |  |
| GCA_900518945.1 | *S. marcescens* |  |  |  |  |  |
| GCA_900518955.1 | *S. marcescens* |  |  |  |  |  |
| GCA_900518975.1 | *S. marcescens* |  |  |  |  |  |
| GCA_902165925.1 | *S. marcescens* | 4928STDY7387874 |  | Faecal |  |  |
| GCA_902166755.1 | *S. marcescens* | 4928STDY7387938 |  | Faecal |  |  |
| GCA_902386095.1 | *S. marcescens* |  |  | Human gut |  |  |
| GCA_902387935.1 | *S. marcescens* |  |  | Human gut |  |  |
| GCA_000521925.1 | *S. marcescens* | BIDMC_44 |  |  |  |  |
| GCA_000521905.1 | *S. marcescens* | BIDMC_50 |  |  |  |  |
| GCA_000465615.2 | *S. marcescens* | EGDHP20 |  | Tannery waste water | India | 2005 |
| GCA_000633335.1 | *S. marcescens* | H1q |  | Carnivorous plant phytotelma | Malaysia |  |
| GCA_000442455.1 | *S. marcescens* | LCTSM166 |  |  |  |  |
| GCA_000264275.1 | *S. marcescens* | LCTSM213 |  |  |  |  |
| GCA_000442375.1 | *S. marcescens* | LCTSM262 |  |  |  |  |
| GCA_000633555.1 | *S. marcescens* | PH1a |  | Carnivorous plant phytotelma | Malaysia |  |
| GCA_000828775.1 | *S. marcescens* | SM39 |  |  |  |  |
| GCA_900029885.1 | *S. marcescens* | SMB2099 |  | Clinical isolate |  |  |
| GCA_000292365.1 | *S. marcescens* | W2.3 |  | Sick tilapia fish | Malaysia |  |
| GCA_000336425.1 | *S. marcescens* | WW4 |  |  |  |  |
| GCA_002007925.2 | *S. marcescens* | 1274 | *Agave sisalana* |  | Brazil | 2010 |
| GCA_001932655.1 | *S. marcescens* | AS1 | *Anopheles stephensi* |  | China | 2013 |
| GCA_001853455.1 | *S. marcescens* | ano1 | *Anopheles stephensi* | Midgut content |  |  |
| GCA_001853495.1 | *S. marcescens* | ano2 | *Anopheles stephensi* | Gut contents |  |  |
| GCA_001889685.1 | *S. marcescens* | sicaria_Ss1_ | *Apis mellifera* | Hemolymph | USA | 2014 |
| GCA_002915435.1 | *S. marcescens* | KZ19 | *Apis mellifera* | Gut | USA | 2016 |
| GCA_002915445.1 | *S. marcescens* | KZ11 | *Apis mellifera* | Gut | USA | 2016 |
| GCA_002915475.1 | *S. marcescens* | KZ2 | *Apis mellifera* | Gut | USA | 2016 |
| GCA_001975745.1 | *S. marcescens* | 19F | *Atelopus zeteki* | Skin | USA | 2016 |
| GCA_002592035.1 | *S. marcescens* | KHCo24B | *Gossypium hirsutum* |  | India | 2015 |
| GCA_000734475.1 | *S. marcescens* | YDC563 | Human | Leg wound | USA | 2012 |
| GCA_000739215.1 | *S. marcescens* | NGSED1015 | Human | Blood | United Kingdom | 2013 |
| GCA_000783615.2 | *S. marcescens* | FDAARGOS_79 | Human | Resp cx | USA | 2013 |
| GCA_000783915.2 | *S. marcescens* | FDAARGOS_65 | Human | Endotracheal aspirate | USA | 2013 |
| GCA_000783975.2 | *S. marcescens* | FDAARGOS_62 | Human | Endotracheal aspirate | USA | 2013 |
| GCA_000805875.1 | *S. marcescens* | RM66262 | Human | Patient with urinary tract infection | Argentina | 2003 |
| GCA_001022215.1 | *S. marcescens* | CAV1492 | Human | Respiratory | USA | 2011 |
| GCA_001030265.1 | *S. marcescens* | BWH57 | Human |  | USA | 2014 |
| GCA_001034375.1 | *S. marcescens* | BWH56 | Human |  | USA | 2014 |
| GCA_001034395.1 | *S. marcescens* | UCI87 | Human |  |  | 2014 |
| GCA_001034405.1 | *S. marcescens* | UCI88 | Human |  |  | 2014 |
| GCA_001060335.1 | *S. marcescens* | 1145_SMAR | Human |  | USA |  |
| GCA_001060585.1 | *S. marcescens* | 1218_SMAR | Human |  | USA |  |
| GCA_001060625.1 | *S. marcescens* | 1241_SMAR | Human |  | USA |  |
| GCA_001060655.1 | *S. marcescens* | 1242_SMAR | Human |  | USA |  |
| GCA_001061145.1 | *S. marcescens* | 1186_SMAR | Human |  | USA |  |
| GCA_001061195.1 | *S. marcescens* | 1198.rep2_SMAR | Human |  | USA |  |
| GCA_001061225.1 | *S. marcescens* | 1219_SMAR | Human |  | USA |  |
| GCA_001062235.1 | *S. marcescens* | 1185_SMAR | Human |  | USA |  |
| GCA_001062285.1 | *S. marcescens* | 1198.rep1_SMAR | Human |  | USA |  |
| GCA_001063125.1 | *S. marcescens* | 276_SMAR | Human |  | USA |  |
| GCA_001063145.1 | *S. marcescens* | 280_SMAR | Human |  | USA |  |
| GCA_001063175.1 | *S. marcescens* | 286_SMAR | Human |  | USA |  |
| GCA_001063325.1 | *S. marcescens* | 410_SMAR | Human |  | USA |  |
| GCA_001063375.1 | *S. marcescens* | 420_SMAR | Human |  | USA |  |
| GCA_001064335.1 | *S. marcescens* | 287_SMAR | Human |  | USA |  |
| GCA_001064345.1 | *S. marcescens* | 290_SMAR | Human |  | USA |  |
| GCA_001064455.1 | *S. marcescens* | 311_SMAR | Human |  | USA |  |
| GCA_001064715.1 | *S. marcescens* | 370_SMAR | Human |  | USA |  |
| GCA_001064725.1 | *S. marcescens* | 374_SMAR | Human |  | USA |  |
| GCA_001064835.1 | *S. marcescens* | 395_SMAR | Human |  | USA |  |
| GCA_001064855.1 | *S. marcescens* | 398_SMAR | Human |  | USA |  |
| GCA_001064975.1 | *S. marcescens* | 454_SMAR | Human |  | USA |  |
| GCA_001065275.1 | *S. marcescens* | 508_SMAR | Human |  | USA |  |
| GCA_001065325.1 | *S. marcescens* | 532_SMAR | Human |  | USA |  |
| GCA_001065845.1 | *S. marcescens* | 666_SMAR | Human |  | USA |  |
| GCA_001065935.1 | *S. marcescens* | 684_SMAR | Human |  | USA |  |
| GCA_001066015.1 | *S. marcescens* | 709_SMAR | Human |  | USA |  |
| GCA_001066945.1 | *S. marcescens* | 698_SMAR | Human |  | USA |  |
| GCA_001067015.1 | *S. marcescens* | 706_SMAR | Human |  | USA |  |
| GCA_001067375.1 | *S. marcescens* | 790_SMAR | Human |  | USA |  |
| GCA_001068085.1 | *S. marcescens* | 907_SMAR | Human |  | USA |  |
| GCA_001076625.1 | *S. marcescens* | 294_SMAR | Human |  | USA |  |
| GCA_001294565.1 | *S. marcescens* | SmUNAM836 | Human | Bronchial aspirate | Mexico | 2005 |
| GCA_001566695.1 | *S. marcescens* | 3691F | Human | Ascitic fluid | Brazil | 2014 |
| GCA_001594385.1 | *S. marcescens* | ICU12a | Human | Host1 | USA | 2014 |
| GCA_001672055.1 | *S. marcescens* | U36365 | Human | Urine | India | 2015 |
| GCA_001909165.1 | *S. marcescens* | SM03 | Human | Stool | India | 2012 |
| GCA_001914155.1 | *S. marcescens* |  | Human | Infant 2 DOL 27 gut | USA | 2013 |
| GCA_002104095.1 | *S. marcescens* | D3 | Human | Bile drainage | USA | 2014 |
| GCA_002104105.1 | *S. marcescens* | D1 | Human | Blood | USA | 2014 |
| GCA_002104115.1 | *S. marcescens* | D2 | Human | Blood | USA | 2014 |
| GCA_002118055.1 | *S. marcescens* | ML2637 | Human | Blood | USA | 2016 |
| GCA_002152845.1 | *S. marcescens* | MGH135 | Human |  | USA | 2015 |
| GCA_002153355.1 | *S. marcescens* | MGH136 | Human |  | USA | 2015 |
| GCA_002220515.1 | *S. marcescens* | UMH2 | Human | University of Michigan Health System | USA | 2014 |
| GCA_002220535.1 | *S. marcescens* | UMH8 | Human | University of Michigan Health System | USA | 2013 |
| GCA_002220555.1 | *S. marcescens* | UMH9 | Human |  | USA | 2014 |
| GCA_002220575.1 | *S. marcescens* | UMH11 | Human | University of Michigan Health System | USA | 22014 |
| GCA_002220595.1 | *S. marcescens* | UMH12 | Human | University of Michigan Health System | USA | 2014 |
| GCA_002220615.1 | *S. marcescens* | UMH1 | Human |  | USA | 2013 |
| GCA_002220635.1 | *S. marcescens* | UMH5 | Human |  | USA | 2014 |
| GCA_002220655.1 | *S. marcescens* | UMH3 | Human | University of Michigan Health System | USA | 2014 |
| GCA_002220675.1 | *S. marcescens* | UMH6 | Human | University of Michigan Health System | USA | 2013 |
| GCA_002220695.1 | *S. marcescens* | UMH10 | Human | University of Michigan Health System | USA | 2014 |
| GCA_002220715.1 | *S. marcescens* | UMH7 | Human |  | USA | 2013 |
| GCA_002250685.1 | *S. marcescens* | at10508 | Human | Lavage | Austria | 2017 |
| GCA_002264105.1 | *S. marcescens* | SE768 | Human | Blood | China | 2014 |
| GCA_002264175.1 | *S. marcescens* | SE4145 | Human | Blood | China | 2015 |
| GCA_002264285.1 | *S. marcescens* | SE3605 | Human | Blood | China | 2015 |
| GCA_002738105.1 | *S. marcescens* | 14ES | Human | Blood culture | Romania | 2012 |
| GCA_002738145.1 | *S. marcescens* | 4TM | Human | Fecal screen | Romania | 2015 |
| GCA_002738155.1 | *S. marcescens* | 7209 | Human | Urine | Romania | 2013 |
| GCA_002738185.1 | *S. marcescens* | 9580 | Human | Urine | Romania | 2015 |
| GCA_002810285.1 | *S. marcescens* | 12TM | Human | Pharyngeal secretions | Romania | 2014 |
| GCA_002886905.1 | *S. marcescens* | YDC1072 | Human |  | USA |  |
| GCA_002887105.1 | *S. marcescens* | YD5092 | Human |  | USA |  |
| GCA_002920335.1 | *S. marcescens* | ID147729 | Human | Sputum | Canada |  |
| GCA_002920345.1 | *S. marcescens* | ID148138 | Human | Blood | Canada |  |
| GCA_002920415.1 | *S. marcescens* | ID148137 | Human | Blood | Canada |  |
| GCA_002920435.1 | *S. marcescens* | ID149855 | Human | Nasal swab | Canada |  |
| GCA_002920455.1 | *S. marcescens* | ID147991 | Human | Eye | Canada |  |
| GCA_002920475.1 | *S. marcescens* | ID149856 | Human | Pus rectal swab | Canada |  |
| GCA_002920485.1 | *S. marcescens* | ID147728 | Human | Sputum | Canada |  |
| GCA_002920515.1 | *S. marcescens* | ID148587 | Human | Surgical wound abdomen | Canada |  |
| GCA_002946295.1 | *S. marcescens* |  | Human | Sputum | China | 2015 |
| GCA_003031545.1 | *S. marcescens* | 95 | Human | Sputum | USA | 2015 |
| GCA_003031645.1 | *S. marcescens* | BWH35 | Human | Sputum | USA | 2012 |
| GCA_003032415.1 | *S. marcescens* | BWH23 | Human | Blood | USA | 2012 |
| GCA_003057465.1 | *S. marcescens* | SRM1 | Human |  | Lebanon | 2017 |
| GCA_003146705.1 | *S. marcescens* | CAV1761 | Human | Perirectal | USA | 2014 |
| GCA_003186475.1 | *S. marcescens* | 332 | Human | Wound | USA | 2016 |
| GCA_003204525.1 | *S. marcescens* | 1756 | Human | Catheter | Brazil | 2013 |
| GCA_003204555.1 | *S. marcescens* | 630 | Human | Blood | Brazil | 2010 |
| GCA_003204565.1 | *S. marcescens* | 642 | Human | Blood | Brazil | 2010 |
| GCA_003204595.1 | *S. marcescens* | 1052 | Human | Tracheal aspirate | Brazil | 2011 |
| GCA_003204615.1 | *S. marcescens* | 1058 | Human | Catheter | Brazil | 2011 |
| GCA_003204635.1 | *S. marcescens* | 1309 | Human | Blood | Brazil | 2012 |
| GCA_003204645.1 | *S. marcescens* | 1763 | Human | Body fluid | Brazil | 2013 |
| GCA_003204675.1 | *S. marcescens* | 1053 | Human | Urine | Brazil | 2011 |
| GCA_003204685.1 | *S. marcescens* | 1057 | Human | Blood | Brazil | 2011 |
| GCA_003204715.1 | *S. marcescens* | 1257 | Human | Urine | Brazil | 2012 |
| GCA_003204725.1 | *S. marcescens* | 1509 | Human | Catheter | Brazil | 2012 |
| GCA_003204755.1 | *S. marcescens* | 1283 | Human | Blood | Brazil | 2012 |
| GCA_003204765.1 | *S. marcescens* | 1299 | Human | Blood | Brazil | 2012 |
| GCA_003204795.1 | *S. marcescens* | 1707 | Human | Blood | Brazil | 2013 |
| GCA_003204805.1 | *S. marcescens* | 4116 | Human | Swab | Brazil | 2014 |
| GCA_003204835.1 | *S. marcescens* | 1056 | Human | Catheter | Brazil | 2011 |
| GCA_003204855.1 | *S. marcescens* | 1703 | Human | Catheter | Brazil | 2013 |
| GCA_003204865.1 | *S. marcescens* | 2031 | Human | Drain | Brazil | 2012 |
| GCA_003204885.1 | *S. marcescens* | 1844 | Human | Swab | Brazil | 2013 |
| GCA_003204905.1 | *S. marcescens* | 1673 | Human | Catheter | Brazil | 2013 |
| GCA_003204935.1 | *S. marcescens* | 2032 | Human | Tap | Brazil | 2012 |
| GCA_003204985.1 | *S. marcescens* | 2039 | Human | Drain | Brazil | 2012 |
| GCA_003204995.1 | *S. marcescens* | 1054 | Human | Urine | Brazil | 2011 |
| GCA_003284885.1 | *S. marcescens* | 1510 | Human | Blood | Brazil | 2012 |
| GCA_003400385.1 | *S. marcescens* | CRE94 | Human | Bloodstream | USA | 2016 |
| GCA_003400545.1 | *S. marcescens* | CRE78 | Human | Bloodstream | USA | 2016 |
| GCA_003400615.1 | *S. marcescens* | CRE72 | Human | Bloodstream | USA | 2016 |
| GCA_003400635.1 | *S. marcescens* | CRE49 | Human | Bloodstream | USA | 2016 |
| GCA_003401215.1 | *S. marcescens* | CRE44 | Human | Bloodstream | USA | 2015 |
| GCA_003402775.1 | *S. marcescens* | CRE35 | Human | Bloodstream | USA | 2015 |
| GCA_003591985.1 | *S. marcescens* | RCE05_sm | Human | Feces | Russia | 2013 |
| GCA_003605765.1 | *S. marcescens* | 3B7 | Human | Stool | Spain | 2016 |
| GCA_003666885.1 | *S. marcescens* | Mex1 | Human |  | Mexico | 2007 |
| GCA_003666905.1 | *S. marcescens* | CHE4 | Human | Urinary infection | Canada | 2004 |
| GCA_003666915.1 | *S. marcescens* | AW | Human | Parotidectomy | Switzerland | 2006 |
| GCA_003666945.1 | *S. marcescens* | S8 | Human |  | United Kingdom | 1982 |
| GCA_003666955.1 | *S. marcescens* | USA1 | Human |  | USA | 2012 |
| GCA_003666965.1 | *S. marcescens* | S6 | Human |  | United Kingdom | 1982 |
| GCA_004109355.1 | *S. marcescens* | 1A0 | Human | Meconium | Spain | 2016 |
| GCA_004179515.1 | *S. marcescens* | ICR003201 | Human | Patient | France | 2016 |
| GCA_004179565.1 | *S. marcescens* | ICR003202 | Human | Patient | France | 2016 |
| GCA_004570635.1 | *S. marcescens* | N2 | Human |  | Egypt | 2017 |
| GCA_006494455.1 | *S. marcescens* | M4 | Human | Catheter tip | Chile | 2014 |
| GCA_006711105.1 | *S. marcescens* | WVU003 | Human | Blood | USA | 2018 |
| GCA_006711125.1 | *S. marcescens* | WVU004 | Human | Blood | USA | 2019 |
| GCA_006711145.1 | *S. marcescens* | WVU005 | Human | Blood | USA | 2019 |
| GCA_006711245.1 | *S. marcescens* | WVU006 | Human | Blood | USA | 2019 |
| GCA_006711405.1 | *S. marcescens* | WVU007 | Human | Blood | USA | 2019 |
| GCA_006711525.1 | *S. marcescens* | WVU008 | Human | Blood | USA | 2019 |
| GCA_006716725.1 | *S. marcescens* | WVU009 | Human | Blood | USA | 2019 |
| GCA_006716825.1 | *S. marcescens* | WVU010 | Human | Blood | USA | 2019 |
| GCA_006838705.1 | *S. marcescens* | WVU001 | Human | Blood | USA | 2018 |
| GCA_006842785.1 | *S. marcescens* | WVU002 | Human | Blood | USA | 2018 |
| GCA_007954045.1 | *S. marcescens* | KCJ3K309 | Human |  | USA | 2019 |
| GCA_007954245.1 | *S. marcescens* | KCJ3K435 | Human |  | USA | 2019 |
| GCA_008180355.1 | *S. marcescens* | KCJ3K308 | Human |  | USA | 2019 |
| GCA_008830745.1 | *S. marcescens* | 188J2 | Human | Rectal swab | France | 2017 |
| GCA_008868645.1 | *S. marcescens* | 2280 | Human | Pulmonary fluid | France | 2019 |
| GCA_000418935.1 | *S. marcescens* | AB42556419isolate1 | Human | Stool sample of individual with | USA |  |
| GCA_000633715.1 | *S. marcescens* | BIDMC_80 | Human | Blood culture |  | 2013 |
| GCA_000633695.1 | *S. marcescens* | BIDMC_81 | Human | Tissue |  | 2013 |
| GCA_000418915.1 | *S. marcescens* | MC458 | Human | Stool sample of individual with | USA |  |
| GCA_000418895.1 | *S. marcescens* | MC459 | Human | Stool sample of individual with | USA |  |
| GCA_000418875.1 | *S. marcescens* | MC460 | Human | Stool sample of individual with | USA |  |
| GCA_000418855.2 | *S. marcescens* | MC6000 | Human | Blood sample of individual with | USA |  |
| GCA_000418835.1 | *S. marcescens* | MC6001 | Human | Blood sample of individual with | USA |  |
| GCA_000418815.1 | *S. marcescens* | MC620 | Human | Stool sample of individual with | USA |  |
| GCA_000342205.1 | *S. marcescens* | VGH107 | Human | Snakebite wound | Taiwan | 2011 |
| GCA_001643155.1 | *S. marcescens* | 092713_C_TSB | Opossum |  | USA | 2013 |
| GCA_000738535.1 | *S. marcescens* | MCB | *Oscheius* sp. MCB | Entomopathogenic nematode Oscheius sp. | South Africa | 2013 |
| GCA_004196355.1 | *S. marcescens* | RPH1 | *Rhodnius prolixus* | Midgut | Brazil | 2016 |
| GCA_004196365.1 | *S. marcescens* | RPA1 | *Rhodnius prolixus* | Midgut | Brazil | 2016 |
| GCA_008122445.1 | *S. marcescens* | C7 | *Sarracenia* | Pitcher plants | USA | 2010 |
| GCA_001280365.1 | *S. marcescens* | RSC14 | *Solanum nigrum* |  | South Korea | 2013 |
| GCA_001756295.1 | *S. marcescens* | TM | termite |  | USA | 2013 |
| GCA_001417865.2 | *S. marcescens* | B3R3 | *Zea mays* |  | China | 2011 |
| GCA_003602305.1 | *S. marcescens* subsp. *marcescens* | 4F |  | Neonatology unit from Hospital | Spain | 1970 |
| GCA_003626775.1 | *S. marcescens* subsp. *marcescens* | 6F |  | Neonatology unit from Hospital | Spain | 1970 |
| GCA_003703735.1 | *S. marcescens* subsp. *marcescens* | 15F |  | Neonatology unit from Hospital | Spain | 1970 |
| GCA_004378065.1 | *S. marcescens* subsp. *marcescens* | 13F |  | Neonatology unit from Hospital | Spain | 1970 |
| GCA_008011855.1 | *S. marcescens* subsp. *marcescens* | S3 |  | Lettuce | Germany | 2015 |
| GCA_008011865.1 | *S. marcescens* subsp. *marcescens* | S5 |  | Carrot | Germany | 2015 |
| GCA_000735445.1 | *S. marcescens* subsp. *marcescens* | ATCC_13880^a^ |  | Pond water |  |  |
| GCA_002899365.1 | *S. marcescens* subsp. *marcescens* | ATCC_13880^a^ |  | Corn polenta | Italy | 1819 |
| GCA_003957875.1 | *S. marcescens* subsp. *marcescens* | ATCC_13880^a^ |  | Pond water | Czech Republic | 1961 |
| GCA_006974205.1 | *S. marcescens* subsp. *marcescens* | ATCC_13880^a^ |  | Pond water | USA | 1969 |
| GCA_000513215.1 | *S. marcescens* subsp. *marcescens* | Db11 |  |  |  |  |
| GCA_001051865.1 | *S. marcescens* subsp. *marcescens* | AH0650_Sm1 | Human | Sputum | Australia | 2014 |
| GCA_001065405.1 | *S. marcescens* subsp. *marcescens* | 546_SSON | Human |  | USA |  |
| GCA_001316425.2 | *S. marcescens* subsp. *marcescens* | 950174583 | Human |  | South Africa |  |
| GCA_001316505.2 | *S. marcescens* subsp. *marcescens* | 950164094 | Human |  | South Africa |  |
| GCA_001316535.2 | *S. marcescens* subsp. *marcescens* | 950172946_950172838 | Human |  | South Africa |  |
| GCA_001316555.2 | *S. marcescens* subsp. *marcescens* | 950196656 | Human |  | South Africa |  |
| GCA_001316595.2 | *S. marcescens* subsp. *marcescens* | 950005607 | Human |  | South Africa |  |
| GCA_001316635.2 | *S. marcescens* subsp. *marcescens* | 9501453777 | Human |  | South Africa |  |
| GCA_001317085.2 | *S. marcescens* subsp. *marcescens* | 946252515 | Human | Tracheal fluid | South Africa | 2013 |
| GCA_001317205.2 | *S. marcescens* subsp. *marcescens* | 945154301 | Human | Urine | South Africa | 2013 |
| GCA_001317285.2 | *S. marcescens* subsp. *marcescens* | 950165859 | Human |  | South Africa |  |
| GCA_001317425.2 | *S. marcescens* subsp. *marcescens* | 945174350 | Human |  | South Africa |  |
| GCA_001317445.2 | *S. marcescens* subsp. *marcescens* | 950166381 | Human |  | South Africa |  |
| GCA_001317455.2 | *S. marcescens* subsp. *marcescens* | 950163360 | Human |  | South Africa |  |
| GCA_001908015.1 | *S. marcescens* subsp. *marcescens* | 189 | Human | Feces | Russia | 2012 |
| GCA_001908035.1 | *S. marcescens* subsp. *marcescens* | 99 | Human |  | Russia | 2009 |
| GCA_003425745.1 | *S. marcescens* subsp. *marcescens* | 163 | Human | Urine | Argentina | 2016 |
| GCA_003605795.1 | *S. marcescens* subsp. *marcescens* | 7B7 | Human | Stool | Spain | 2016 |
| GCA_004109385.1 | *S. marcescens* subsp. *marcescens* | 6B0 | Human | Meconium | Spain | 2016 |
| GCA_002762595.1 | *S. marcescens* subsp. *sakuensis* | K27 |  | Marine |  |  |
| GCA_003428265.1 | *S. marcescens* subsp. *sakuensis* | KCTC_42172 |  |  |  |  |
| GCA_004684145.1 | *S. marcescens* subsp. *sakuensis* | DSM_17174 |  | Activated sludge | Japan | 1998 |
| GCA_007280475.1 | *S. marcescens* subsp. *sakuensis* | S11 |  | Cucumber | Germany | 2015 |
| GCA_008011745.1 | *S. marcescens* subsp. *sakuensis* | S16 |  | Carrot | Germany | 2015 |
| GCA_008011755.1 | *S. marcescens* subsp. *sakuensis* | S14 |  | Marjoram | Germany | 2015 |
| GCA_008011775.1 | *S. marcescens* subsp. *sakuensis* | S12 |  | Cucumber | Germany | 2015 |
| GCA_002082115.1 | *S. nematodiphila* | MB307 |  | Rhizosphere of Cannabis sativa | Pakistan | 2013 |
| GCA_008011815.1 | *S. nematodiphila* | S8 |  | Cucumber | Germany | 2015 |
| GCA_900101535.1 | *S. nematodiphila* | CGMCC_1.6853^a^ |  |  |  |  |
| GCA_002185265.2 | *S. nematodiphila* | CRK0003 | Human | Blood | USA | 2015 |
| GCA_000738675.1 | *S. nematodiphila* DZ0503SBS1 | DSM_21420^a^ | *Heterorhabditidoides chongmingensis* | Intestine | China | 2008 |
| GCA_002082525.1 | S. *nematodiphila* DZ0503SBS1 | DZ0503SBS1^a^ | *Heterorhabditidoides chongmingensis* | Biological sample | China | 2009 |
| GCA_900005125.1 | *S. nematodiphila* WCU338 | WCU338 |  |  |  |  |
| GCA_003576035.1 | *S. odorifera* | KCTC_2937 |  |  |  |  |
| GCA_900635445.1 | *S. odorifera* | NCTC11214 |  | Not available to be reported later |  |  |
| GCA_000163595.1 | *S. odorifera* | DSM_4582^a^ |  |  |  |  |
| GCA_002206385.2 | *S. odorifera* | FDAARGOS_353 | Human | Sputum | France | 1978 |
| GCA_001976145.1 | *S. oryzae* | J116 | rice |  | China | 2015 |
| GCA_000648575.1 | *S. plymuthica* | V4 |  | Milk processing plant | Portugal | 2006 |
| GCA_001590765.1 | *S. odorifera* | HROC48 |  | Rhizosphere of Brassica napus | Germany |  |
| GCA_001896215.1 | *S. odorifera* | tumat |  | Plant debris in the intestinal content | Russia | 2011 |
| GCA_003206015.1 | *S. odorifera* | 4Rx5 |  | Soil adhering to oilseed rape's roots | Germany | 1999 |
| GCA_003634415.1 | *S. odorifera* | WS3236 |  |  |  |  |
| GCA_900478125.1 | *S. odorifera* | NCTC12961 |  |  |  |  |
| GCA_900635625.1 | *S. odorifera* | NCTC8900 |  | Not available to be reported later |  |  |
| GCA_900637965.1 | *S. odorifera* | NCTC8015 |  | Canal water |  |  |
| GCA_000176835.2 | *S. odorifera* | 4Rx13 |  |  |  |  |
| GCA_000214235.1 | *S. odorifera* | AS9 |  |  |  |  |
| GCA_001590925.1 | *S. odorifera* | NBRC_102599^a^ |  |  |  |  |
| GCA_000261045.2 | *S. odorifera* | PRI2c |  | Maize rhizosphere soil | Netherlands | 2004 |
| GCA_000478545.1 | *S. odorifera* | RVH1 |  |  |  |  |
| GCA_000438825.1 | *S. odorifera* | S13 |  | Styrian pumpkin anthrosphere |  |  |
| GCA_001663115.1 | *S. odorifera* | 3Rp8 | *Brassica napus* | Organic material | Germany | 1998 |
| GCA_000300895.1 | *S. odorifera* | A30 | potato | Potato tuber rotten tissue | Netherlands | 2009 |
| GCA_001663135.1 | *S. odorifera* | 3Re418 | *Solanum tuberosum L. cv.* Cilena | Organic material | Germany | 2001 |
| GCA_001606165.1 | *S. odorifera* | A153 | *Triticum aestivum* | Rhizosphere soil | Sweden | 1987 |
| GCA_003699145.1 | *S. odorifera* | ICMP_9395 | *Triticum aestivum* |  | Russia |  |
| GCA_004153725.1 | *S. proteamaculans* | B41156 |  | Ground beef | USA | 2016 |
| GCA_004153735.1 | *S. proteamaculans* | B41162 |  | Ground beef | USA | 2016 |
| GCA_004153785.1 | *S. proteamaculans* | B59510 |  |  |  | 2016 |
| GCA_004684015.1 | *S. proteamaculans* | D2.2 |  | Mining waste | Australia | 2016 |
| GCA_008830365.1 | *S. proteamaculans* | CCUG_14510^a^ |  |  |  |  |
| GCA_900175135.1 | *S. proteamaculans* |  |  | Organic beef carpaccio |  | 2009 |
| GCA_900457085.1 | *S. proteamaculans* | NCTC10861 |  |  |  |  |
| GCA_000018085.1 | *S. proteamaculans* | 568 |  |  |  |  |
| GCA_004217345.1 | *S. quinivorans* | 124R |  |  | USA |  |
| GCA_004684265.1 | *S. quinivorans* | PKL_12 |  | Rhizospheric soil of Picrorrhiza kurroa | India | 2015 |
| GCA_900456965.1 | *S. quinivorans* | NCTC13194 |  |  |  |  |
| GCA_900457005.1 | *S. quinivorans* | NCTC13189 |  |  |  |  |
| GCA_900457075.1 | *S. quinivorans* | NCTC11544^a^ |  |  |  |  |
| GCA_900638135.1 | *S. quinivorans* | NCTC13188 |  |  |  |  |
| GCA_900478395.1 | *S. rubidaea* | NCTC10848 |  |  |  |  |
| GCA_900635665.1 | *S. rubidaea* | NCTC9419 |  |  |  |  |
| GCA_900638005.1 | *S. rubidaea* | NCTC10036 |  | Finger |  |  |
| GCA_901472405.1 | *S. rubidaea* | NCTC12971^a^ |  |  |  |  |
| GCA_001598675.1 | *S. rubidaea* | NBRC_103169^a^ |  |  |  |  |
| GCA_001304675.1 | *S. rubidaea* | CIP_103234^a^ | Human |  | France | 1980 |
| GCA_001572725.1 | *S. rubidaea* | 1122 | Human | Sputum | China | 2014 |
| GCA_003540595.1 | *Serratia* sp. |  |  | Metagenome |  |  |
| GCA_001537385.1 | *Serratia* sp. | 2880STDY5682894 |  | Blood | United Kingdom | 2005 |
| GCA_001537265.1 | *Serratia* sp. | 2880STDY5682895 |  | Blood | United Kingdom | 2005 |
| GCA_000463345.3 | *Serratia* sp. | ATCC_39006 |  | Cheesequake salt marsh new jersey | USA | 1982 |
| GCA_002847015.1 | *Serratia* sp. | ATCC_39006 |  |  |  | 2000 |
| GCA_004361715.1 | *Serratia* sp. | BIGb0156 |  |  |  |  |
| GCA_002607755.1 | *Serratia* sp. | BW106 |  | Cheese rind | France | 2011 |
| GCA_000336365.1 | *Serratia* sp. | C1 |  |  |  |  |
| GCA_000330865.1 | *Serratia* sp. | FGI94 |  |  |  |  |
| GCA_000695995.1 | *Serratia* sp. | FS14 |  |  |  |  |
| GCA_000392415.2 | *Serratia* sp. | GLFA |  | Oil contaminated soil | USA | 2011 |
| GCA_000633315.1 | *Serratia* sp. | H1n |  | Carnivorous plant phytotelma | Malaysia |  |
| GCA_000633355.1 | *Serratia* sp. | H1w |  | Carnivorous plant phytotelma | Malaysia |  |
| GCA_001714765.1 | *Serratia* sp. | ISTD04 |  | Marble rocks of palaeoproterozoic | India | 2009 |
| GCA_900215455.1 | *Serratia* sp. | JKS000199 |  |  |  |  |
| GCA_900215445.1 | *Serratia* sp. | JKS296 |  |  |  |  |
| GCA_900007715.1 | *Serratia* sp. | LCN16 |  |  |  |  |
| GCA_002966855.1 | *Serratia* sp. | MYb239 |  | Compost | Germany |  |
| GCA_003097335.1 | *Serratia* sp. | S1B |  | Soil | USA | 2017 |
| GCA_000347995.1 | *Serratia* sp. | S4 |  | Rhizosphere of naturally growing | Sweden | 1980 |
| GCA_003591175.1 | *Serratia* sp. | S40 |  | Potato | Denmark | 2016 |
| GCA_008011825.1 | *Serratia* sp. | S9 |  | Cucumber | Germany | 2015 |
| GCA_000747565.1 | *Serratia* sp. | SCBI |  |  |  |  |
| GCA_006517585.1 | *Serratia* sp. | SRS8S2018 |  | Mercury contaminated soil collected | USA | 2018 |
| GCA_002935055.1 | *Serratia* sp. | SSNIH1 |  |  | USA | 2015 |
| GCA_002920145.1 | *Serratia* sp. | SSNIH2 |  |  | USA | 2015 |
| GCA_002920115.1 | *Serratia* sp. | SSNIH3 |  |  | USA | 2016 |
| GCA_002918955.1 | *Serratia* sp. | SSNIH4 |  |  | USA | 2016 |
| GCA_002920095.1 | *Serratia* sp. | SSNIH5 |  |  | USA | 2016 |
| GCA_001011075.1 | *Serratia* sp. | TEL |  | Soil | South Africa | 2014 |
| GCA_002797215.1 | *Serratia* sp. | TKO39 |  | Pond water sludge |  | 2012 |
| GCA_001642805.2 | *Serratia* sp. | YD25^a^ |  | Rhizosphere soil | China | 2011 |
| GCA_000743355.1 | *Serratia* sp. | Ag1 | *Anopheles gambiae* | Midgut of the mosquito Anopheles | France | 2014 |
| GCA_000743365.1 | *Serratia* sp. | Ag2 | *Anopheles gambiae* | Mosquito larval gut | USA | 2014 |
| GCA_001422565.1 | *Serratia* sp. | Leaf50 | *Arabidopsis thaliana* | Leaf | Switzerland | 2013 |
| GCA_001422575.1 | *Serratia* sp. | Leaf51 | *Arabidopsis thaliana* | Leaf | Switzerland | 2013 |
| GCA_000257645.1 | *Serratia* sp. | M24T3 | *Bursaphelenchus xylophilus* |  | Portugal | 2009 |
| GCA_003668775.1 | *Serratia* sp. | 3ACOL1 | *Cerambycidae* sp. | Larvae | Norway | 2017 |
| GCA_003691565.1 | *Serratia* sp. | P2ACOL2 | *Cerambycidae* sp. | Larvae | Norway | 2017 |
| GCA_000496755.2 | *Serratia* sp. | DD3 | *Daphnia magna* | Gut | Germany | 2008 |
| GCA_003641105.1 | *Serratia* sp. | 1D1416 | *Euonymus japonicus* | Gall tissue |  | 1972 |
| GCA_001076875.1 | *Serratia* sp. | 506_PEND | Human |  | USA |  |
| GCA_003812745.1 | *Serratia* sp. | FDAARGOS_506 | Human | Endotracheal aspirate |  | 2015 |
| GCA_001808215.1 | *Serratia* sp. | HMSC15F11 | Human |  |  |  |
| GCA_002752475.1 | *Serratia* sp. | OLAL2 | *Orius laevigatus* | Lab maintained population of field | Greece |  |
| GCA_002752085.1 | *Serratia* sp. | OLBL1 | *Orius laevigatus* | Lab maintained population of field | Spain |  |
| GCA_002752255.1 | *Serratia* sp. | OLCL1 | *Orius laevigatus* | Lab maintained population of field | Spain |  |
| GCA_002752235.1 | *Serratia* sp. | OLDL1 | *Orius laevigatus* | Whole insect macerate from a lab | Italy |  |
| GCA_002752095.1 | *Serratia* sp. | OLEL1 | *Orius laevigatus* | Lab maintained population of field | Italy |  |
| GCA_002752135.1 | *Serratia* sp. | OLFL2 | *Orius laevigatus* | Whole insect macerate from a lab | Spain |  |
| GCA_002752155.1 | *Serratia* sp. | OLHL2 | *Orius laevigatus* | Lab maintained population of field | Spain |  |
| GCA_002752265.1 | *Serratia* sp. | OLIL2 | *Orius laevigatus* | Lab maintained population of field | Spain |  |
| GCA_002752195.1 | *Serratia* sp. | OLJL1 | *Orius laevigatus* | Lab maintained population of field | Spain |  |
| GCA_002751955.1 | *Serratia* sp. | OLLOLW30 | *Orius laevigatus* | Lab maintained population of field | Spain |  |
| GCA_002751935.1 | *Serratia* sp. | OLMTLW26 | *Orius laevigatus* | Whole insect macerate from a laboratory | Spain |  |
| GCA_002752455.1 | *Serratia* sp. | OMLW3 | *Orius majusculus* | Lab maintained population of field | Spain |  |
| GCA_002752435.1 | *Serratia* sp. | OSPLW9 | *Orius niger* | Lab maintained population of field | Switzerland |  |
| GCA_002752535.1 | *Serratia* sp. | OPWLW2 | *Orius* sp. | Field collected specimens | Spain |  |
| GCA_002752495.1 | *Serratia* sp. | OPWLW3 | *Orius* sp. | Field collected specimens | Spain |  |
| GCA_003719595.1 | *Serratia* sp. | LS1 | *Orthaga achatina* |  |  | 2016 |
| GCA_001984565.1 | *Serratia* sp. | S119 | peanut | Nodule | Argentina | 2007 |
| GCA_000214195.1 | *Serratia* sp. | AS12 | rapeseed plant |  |  |  |
| GCA_000214805.1 | *Serratia* sp. | AS13 | rapeseed plant |  |  |  |
| GCA_001692375.1 | *Serratia* sp. | 142641 | rose | Plant |  | 2014 |
| GCA_003028435.1 | *Serratia* sp. | Nf2 | Saccharum hybrid cultivar |  | Colombia | 2014 |
| GCA_000821185.1 | *S. symbiotica* | type_strain_CWBI2.3^a^ |  |  |  |  |
| GCA_900155695.1 | *S. symbiotica* | SCifornacula |  | Whole body |  | 2010 |
| GCA_900380265.1 | *S. symbiotica* | SeCistrobi |  | Host's whole body |  | 2012 |
| GCA_008370165.1 | *S. symbiotica* | IS | *Acyrthosiphon pisum* | Haemolymph |  |  |
| GCA_000186485.2 | *S. symbiotica* | Tucson | *Acyrthosiphon pisum* pea aphid |  |  |  |
| GCA_000238975.1 | *S. symbiotica* | Cinara_cedri | *Cinara cedri* |  |  |  |
| GCA_900002265.1 | *S. symbiotica* | SCtVLC | *Cinara tujafilina* |  | Spain |  |
| GCA_900016775.1 | *S. symbiotica* | STs | *Tuberolachnus salignus* |  |  | 2013 |
| GCA_000988045.1 | *S. ureilytica* | Lr5_4 |  | Geothermal spring water | Chile | 2011 |
| GCA_008011725.1 | *S. ureilytica* | S17 |  | Carrot | Germany | 2015 |
| GCA_008011875.1 | *S. ureilytica* | S6 |  | Lettuce | Germany | 2015 |
| GCA_002844245.1 | *S. ureilytica* | DW2 | *Codonopsis pilosula* | Rhizosphere |  |  |
| GCA_902207215.1 | *S. vespertilionis* |  |  |  |  |  |

^a^ *Serratia* spp. type strains as listed in List of Prokaryotic names with Standing in Nomenclature (LPSN) [1]

**References**

1. Parte AC, Sardà Carbasse J, Meier-Kolthoff JP, Reimer LC, Göker M. List of Prokaryotic names with Standing in Nomenclature (LPSN) moves to the DSMZ. International Journal of Systematic and Evolutionary Microbiology. 2020;70(11):5607-12. doi: <https://doi.org/10.1099/ijsem.0.004332>.
