# Supplementary material for: Genomic characterisation of an entomopathogenic strain of Serratia ureilytica in the critically endangered phasmid Dryococelus australis
Source: PLoS One. 2022 Apr 20;17(4):e0265967. doi: 10.1371/journal.pone.0265967 (PMC9020675; doi:10.1371/journal.pone.0265967)
Supplement: S3 Table — (DOCX) [file pone.0265967.s007.docx]

**S3 Table. *Serratia* spp*.* isolated from the insect environment (water, nest boxes, frass and floor samples).**

| Sample type | No. samples collected | No. colonies tested | No. of confirmed  *Serratia* spp. isolates | Isolate name^a^ | Pigmentation |
| --- | --- | --- | --- | --- | --- |
| Drinking water | 2 | 20 | 1 | 5W2w | Non-pigmented |
| Floor surface | 1 | 20 | 1 | 5F1r | Pigmented |
| Frass | 6 | 60 | 0 | - | - |
| Nest box surface | 6 | 0 | 0 | - | - |
| Drinking water | 2 | 20 | 1 | 6W2r | Pigmented |
| Floor surface | 1 | 23 | 2 | 6F1w | Non-pigmented |
|  |  |  |  | 6F1r | Pigmented |
| Frass | 4 | 40 | 1 | 6Fr4r | Pigmented |
| Nest box surface | 4 | 13 | 1 | 6B1r | Pigmented |

^a^ Sample naming convention reflects Glass house number (5 and 6), sample type (W, water; F, floor; Fr, frass; B, nest box), sample number and pigmentation (w, white; r, red)
