# Supplementary material for: Genomic characterisation of an entomopathogenic strain of Serratia ureilytica in the critically endangered phasmid Dryococelus australis
Source: PLoS One. 2022 Apr 20;17(4):e0265967. doi: 10.1371/journal.pone.0265967 (PMC9020675; doi:10.1371/journal.pone.0265967)
Supplement: S4 Table — (DOCX) [file pone.0265967.s008.docx]

**S4 Table. Histopathological analysis of a subset of 25 dead insects.**

| Animal ID | Haemolymph culture | Gross Plaques | Head^a^ | Limb | Fat body^a^ | Gonad | Malpighian tubules | Foregut (FG) | FG/MG junction | Midgut (MG) | Hindgut/MG junction | Rectum/cloaca |
| --- | --- | --- | --- | --- | --- | --- | --- | --- | --- | --- | --- | --- |
| 3 | *Serratia* sp. | None | 2 | 0 | 2 | 0 | 0 | 0 | 3 | 0 | 0 | 0 |
| 4 | *Serratia* sp. | Diffuse fat body brown discolouration | 2 | 2 | 2 | 0 | 3 | 0 | 0 | 2 | 0 | 0 |
| 11 | *Serratia* sp. | Focal discolouration fat body | 2 | 2 | 2 | 1 | 2 | 0 | 0 | 0 | 0 | 3 |
| 12 | *Serratia* sp. | Diffuse fat body brown discolouration | 1 | 1 | 0 | 0 | 0 | 0 | 3 | 0 | 0 | 2 |
| 13 | *Serratia* sp. | None | 2 | 1 | 2 | 2 | 2 | 0 | 0 | 0 | 0 | 0 |
| 16 | *Serratia* sp. | Focal discolouration fat body | 2 | 0 | 3 | 0 | 0 | 0 | 1 | 0 | 0 | 0 |
| 18 | *Serratia* sp. | None | 1 | 0 | 1 | 2 | 0 | 0 | 0 | 0 | 0 | 0 |
| 19 | *Serratia* sp. | Diffuse fat body brown discolouration | 3 | 2 | 2 | 0 | 0 | 3 | 0 | 0 | 0 | 0 |
| 22 | *Serratia* sp. | Diffuse fat body brown discolouration | 3 | 2 | 3 | 3 | 0 | 0 | 0 | 1 | 0 | 0 |
| 23 | *Serratia* sp. | None | 2 | 2 | 2 | 2 | 0 | 0 | 0 | 0 | 0 | 0 |
| 1 | *Pseudomonas aeruginosa* | None | 2 | 2 | 1 | 3 | 0 | 0 | 0 | 0 | 0 | 0 |
| 2 | *Pseudomonas aeruginosa* | None | 1 | 2 | N/A | N/A | 0 | 0 | 0 | 0 | 0 | 0 |
| 7 | *Pseudomonas aeruginosa* | None | 1 | 0 | 1 | 0 | 0 | 0 | 0 | 0 | 0 | 0 |
| 24 | *Pseudomonas aeruginosa* | None | 0 | 0 | 0 | 0 | 0 | 0 | 3 | 0 | 0 | 1 |
| 30 | *Pseudomonas aeruginosa* | None | 2 | 2 | 2 | 3 | 0 | 0 | 0 | 0 | 0 | 3 |
| 52 | *Pseudomonas aeruginosa* | None | 0 | 0 | 0 | 3 | 1 | 0 | 0 | 0 | 0 | 0 |
| 56 | *Pseudomonas aeruginosa* | None | 0 | 1 | 0 | 3 | 0 | 0 | 0 | 0 | 0 | 0 |
| 58 | *Proteus vulgaris* | Pigment at MG/FG equivocal | 3 | N/A | 2 | 3 | 0 | 0 | 0 | 0 | 0 | 0 |
| 20 | *Acinetobacter vivianii* | fat body firm to chalky | 0 | N/A | 0 | 0 | 0 | 0 | 0 | 0 | 0 | 0 |
| 29 | *Escherichia coli* | None | 2 | 0 | 0 | 3 | 0 | 0 | 0 | 0 | 0 | 0 |
| 15 | mixed, unknown | None | 0 | 0 | 0 | 0 | 0 | 0 | 0 | 0 | 0 | 0 |
| 25 | mixed, unknown | None | 0 | 0 | 3 | 0 | 0 | 0 | 0 | 0 | 0 | 0 |
| 8 | no growth | None | 0 | 0 | 0 | 0 | 0 | 0 | 0 | 0 | 0 | 0 |
| 27 | no growth | None | 2 | 0 | 0 | 0 | 3 | 2 | 0 | 0 | 0 | 0 |
| 57 | no growth | Foregut and midgut empty | 0 | 2 | 0 | 0 | 0 | 0 | 0 | 0 | 0 | 0 |

^a^ At P < 0.05, the variance of the lesion scores for the head and fat body of insects where *Serratia* sp. was isolated were significantly different from the lesion scores of insects where no significant growth was recorded. The Kruskal-Wallis test was used for this analysis.
