# Supplementary material for: Genomic characterisation of an entomopathogenic strain of Serratia ureilytica in the critically endangered phasmid Dryococelus australis
Source: PLoS One. 2022 Apr 20;17(4):e0265967. doi: 10.1371/journal.pone.0265967 (PMC9020675; doi:10.1371/journal.pone.0265967)
Supplement: S5 Table — (DOCX) [file pone.0265967.s009.docx]

**S5 Table. Summary of PFGE pulsotypes and rep-PCR profiles of *Serratia spp* isolated from insect haemolymph and the insect environment.**

| Isolate | Origin | Date of sample collection | Pulsotype | rep-PCR profile related/unrelated to AM923 |
| --- | --- | --- | --- | --- |
| AM1003 | haemolymph | 03/10/2014 | A | ND |
| VW347 ^a^ | haemolymph | 06/02/2014 | A1 | ND |
| JU810n1 | haemolymph | 10/08/2014 | A1 | ND |
| AF820 | haemolymph | 20/08/2014 | A1 | ND |
| JU820 | haemolymph | 20/08/2014 | A1 | ND |
| JF824a | haemolymph | 24/08/2014 | A1 | ND |
| SM911 ^c^ | haemolymph | 10/09/2014 | A1 | ND |
| AM923 ^b^ | haemolymph | 23/09/2014 | A1 | - |
| JM925 | haemolymph | 05/09/2014 | A1 | ND |
| AF925 | haemolymph | 25/09/2014 | A1 | ND |
| AF1006 ^c^ | haemolymph | 06/10/2014 | A1 | ND |
| AF1016 | haemolymph | 16/10/2014 | A1 | ND |
| AM1023 | haemolymph | 23/10/2014 | A1 | ND |
| 6F1w | environmental | 2/2015 | A1 | ND |
| 5W2w | environmental | 2/2015 | A1 | ND |
| AF828 ^c^ | haemolymph | 28/08/2014 | A2 | ND |
| JU830 | haemolymph | 30/08/2014 | A2 | ND |
| JU929 ^c^ | haemolymph | 29/09/2014 | A2 | ND |
| AF1007 ^c^ | haemolymph | 07/10/2014 | A2 | ND |
| AF1008 ^c^ | haemolymph | 08/10/2014 | A2 | ND |
| AF1013 | haemolymph | 13/10/2014 | A2 | ND |
| AF1020 ^c^ | haemolymph | 20/10/2014 | A2 | ND |
| AF1021 | haemolymph | 21/10/2014 | A2 | ND |
| VW348 ^a^ | haemolymph | 06/02/2014 | B1 | ND |
| SM1025 ^c^ | haemolymph | 25/10/2014 | B1 | ND |
| AM1004^b^ | haemolymph | 04/10/2014 | B1 | ND |
| 6B1r | environmental | 2/2015 | B2 | ND |
| 6Fr4r | environmental | 2/2015 | B2 | ND |
| 6W2r | environmental | 2/2015 | B2 | ND |
| Toronto zoo ^a^ | haemolymph | 2/2016 | ND | related |
| Bristol zoo ^a^ | haemolymph | 9/2017 | ND | unrelated |
| 2017-042 | haemolymph | 18/01/2017 | ND | related |
| 2017-065 | haemolymph | 25/01/2017 | ND | related |
| 2017-249 | haemolymph | 09/03/2017 | ND | related |
| 2019-015 | haemolymph | 10/01/2019 | ND | related |

^a^ Isolates provided by Zoos Victoria for this study

^b^ Representative isolate of pulsotype selected for whole genome sequence analysis

^c^ Histopathological analysis conducted
