# Supplementary material for: Genomic characterisation of an entomopathogenic strain of Serratia ureilytica in the critically endangered phasmid Dryococelus australis
Source: PLoS One. 2022 Apr 20;17(4):e0265967. doi: 10.1371/journal.pone.0265967 (PMC9020675; doi:10.1371/journal.pone.0265967)
Supplement: S6 Table — (DOCX) [file pone.0265967.s010.docx]

**S6 Table. Fast ANI analysis to compare strains AM923 and AM1004 with reference strains of *Serratia* spp..**

| Reference Genome | ANI value (%)  (Count of bidirectional mapping fragments) | |
| --- | --- | --- |
|  | Strain AM923 | Strain AM1004 |
| *Serratia ficaria* NCTC12148 | 88.62 (1363) | 88.83 (1333) |
| *Serratia fonticola* DSM4576 | 82.41 (981) | 82.38 (995) |
| *Serratia grimesii* NBRC13537 | 82.94 (1094) | 83.10 (1111) |
| *Serratia liquefaciens* SER00158 | 84.71 (1273) | 84.58 (1246) |
| *Serratia marcescens* SCQ1 | 95.28 (1492) | **95.09 (1482)** |
| *Serratia nematodiphila* DH-S01 | 95.19 (1543) | 94.88 (1512) |
| *Serratia odorífera* FDAARGOS 353 | 83.38 (1074) | 83.38 (1074) |
| *Serratia oryzae* J11-6 | 82.27 (898) | 82.45 (896) |
| *Serratia plymuthica* AS9 | 85.10 (1282) | 85.15 (1240) |
| *Serratia proteamaculans* CCUG 14510 | 84.25 (1232) | 84.30 (1232) |
| *Serratia rubidae* FDAARGOS 926 | 84.66 (1126) | 84.63 (1100) |
| *Serratia surfactantfaciens* YD25 | 93.99 (1513) | 94.41 (1518) |
| *Serratia symbiotica* Apa8A1 | 81.77 (648) | 81.80 (638) |
| *Serratia ureilytica* SER00211 | **99.00 (1615)** | 94.52 (1524) |
