# Supplementary material for: Genomic characterisation of an entomopathogenic strain of Serratia ureilytica in the critically endangered phasmid Dryococelus australis
Source: PLoS One. 2022 Apr 20;17(4):e0265967. doi: 10.1371/journal.pone.0265967 (PMC9020675; doi:10.1371/journal.pone.0265967)
Supplement: S7 Table — (DOCX) [file pone.0265967.s011.docx]

**S7 Table. CDSs present in all insect isolates from clade A and fewer than 20% of isolates from clades B and C.**

| Gene | Start | Stop | Strand | Annotation | clade A % | clade B % | clade C % | Others % |
| --- | --- | --- | --- | --- | --- | --- | --- | --- |
| *ibp*B | 11878 | 12306 |  | Small heat shock protein IbpB | 100 | 0 | 0 | 1 |
| *yia*D | 27921 | 28583 | c | Putative lipoprotein YiaD | 98 | 6 | 19 | 29 |
| *yja*B | 28832 | 29284 | c | Putative N-acetyltransferase YjaB | 100 | 18 | 19 | 14 |
| *esa*R | 32626 | 33372 |  | Transcriptional activator protein EsaR | 100 | 0 | 10 | 4 |
| *esa*I | 33319 | 33993 | c | Acyl-homoserine-lactone synthase | 100 | 0 | 10 | 3 |
| *tsr* | 372255 | 373880 |  | Methyl-accepting chemotaxis protein I | 80 | 4 | 4 | 7 |
| ***phe*A2** | **471094** | **471636** |  | **Secreted chorismate mutase** | **98** | **0** | **1** | **1** |
| *ssu*D | 542933 | 544078 |  | Alkanesulfonate monooxygenase | 94 | 17 | 7 | 1 |
| *atz*E | 903393 | 904787 | c | Biuret hydrolase | 100 | 19 | 0 | 2 |
| *fli*Y | 907785 | 908576 |  | L-cystine-binding protein FliY | 98 | 19 | 0 | 42 |
| *gln*M | 908589 | 909254 |  | Putative glutamine ABC transporter permease protein GlnM | 100 | 19 | 0 | 43 |
| *yec*S | 909254 | 909910 |  | L-cystine transport system permease protein YecS | 100 | 19 | 0 | 43 |
| *gln*Q | 909891 | 910634 |  | Glutamine transport ATP-binding protein GlnQ | 100 | 19 | 0 | 44 |
| ***ure*A2** | **919995** | **920297** |  | **Urease subunit gamma 2** | **100** | **16** | **9** | **4** |
| ***ure*B** | **920353** | **920799** |  | **Urease subunit beta** | **100** | **16** | **9** | **2** |
| ***ure*C** | **920803** | **922518** |  | **Urease subunit alpha** | **100** | **16** | **9** | **4** |
| ***ure*E** | **922531** | **923127** |  | **Urease accessory protein UreE** | **100** | **16** | **9** | **2** |
| ***ure*F** | **923124** | **923810** |  | **Urease accessory protein UreF** | **100** | **16** | **9** | **2** |
| ***ure*G** | **923830** | **924465** |  | **Urease accessory protein UreG** | **98** | **16** | **9** | **4** |
| ***ure*D** | **924462** | **925409** |  | **Urease accessory protein UreD** | **100** | **16** | **9** | **2** |
| **utp** | **925429** | **926406** |  | **Urea transporter** | **96** | **16** | **9** | **2** |
| *hox*N | 926447 | 927490 |  | High-affinity nickel transport protein | 100 | 16 | 9 | 2 |
| *hcn*B | 1930390 | 1931658 |  | Hydrogen cyanide synthase subunit HcnB | 100 | 0 | 0 | 1 |
| *bet*A | 1943431 | 1945056 | c | Oxygen-dependent choline dehydrogenase | 98 | 0 | 7 | 1 |
| *dml*R | 1945165 | 1946091 | c | HTH-type transcriptional regulator DmlR | 98 | 0 | 7 | 1 |
| *mac*A | 1951412 | 1952602 | c | Macrolide export protein MacA | 96 | 0 | 7 | 1 |
| *xyp*A | 2047318 | 2048247 |  | Xylitol-binding protein | 96 | 4 | 5 | 2 |
| *rox*A | 2107465 | 2108586 | c | 50S ribosomal protein L16 3-hydroxylase | 82 | 8 | 13 | 2 |
| *pho*P | 2110134 | 2110805 | c | Transcriptional regulatory protein PhoP | 82 | 19 | 19 | 10 |
| *ygd*R | 2129233 | 2129466 | c | Putative lipoprotein YgdR | 92 | 0 | 0 | 1 |
| *btu*F | 2226640 | 2227773 |  | Vitamin B12-binding protein | 92 | 3 | 9 | 2 |
| *sot*B | 2436040 | 2437239 | c | Sugar efflux transporter | 100 | 19 | 1 | 2 |
| *aco*R | 2445295 | 2447070 |  | Acetoin catabolism regulatory protein | 98 | 4 | 0 | 1 |
| ***qui*A** | **2463192** | **2465627** | **c** | **Quinate/shikimate dehydrogenase (quinone)** | **98** | **0** | **0** | **12** |
| *tet*A | 2583728 | 2584894 |  | Tetracycline resistance protein, class C | 96 | 14 | 1 | 2 |
| *pra*I | 2588672 | 2589850 | c | 4-hydroxybenzoate 3-monooxygenase (NAD(P)H) | 94 | 13 | 1 | 5 |
| *stt*H | 2616463 | 2617008 |  | Streptothricin hydrolase | 96 | 18 | 5 | 3 |
| *yof*A | 2794866 | 2795720 | c | HTH-type transcriptional regulator YofA | 96 | 0 | 0 | 1 |
| *dxr* | 2798665 | 2799699 |  | D-xylulose reductase | 96 | 14 | 13 | 8 |
| *xyl*B | 2799704 | 2801221 |  | Xylulose kinase | 96 | 17 | 13 | 8 |
| *gap*A | 2845090 | 2846085 |  | Glyceraldehyde-3-phosphate dehydrogenase A | 98 | 6 | 18 | 40 |
| *dad*X | 2857562 | 2858635 | c | Alanine racemase, catabolic | 94 | 0 | 0 | 1 |
| *tua*G | 3030894 | 3031637 | c | Putative teichuronic acid biosynthesis glycosyltransferase TuaG | 98 | 0 | 0 | 38 |
| *pgl*H | 3031648 | 3032703 | c | GalNAc-alpha-(1->4)-GalNAc-alpha-(1->3)- diNAcBac-PP-undecaprenol alpha-1,4-N-acetyl-D-galactosaminyltransferase | 98 | 0 | 0 | 1 |
| *pca*B | 3326751 | 3328100 |  | 3-carboxy-cis,cis-muconate cycloisomerase | 100 | 2 | 0 | 1 |
| *yod*B | 3693404 | 3693928 | c | Cytochrome b561 | 82 | 14 | 8 | 7 |
| *srl*R | 4241942 | 4242760 | c | Glucitol operon repressor | 98 | 11 | 0 | 6 |
| ***fdh*S** | **4462577** | **4463149** |  | **Fructose dehydrogenase small subunit** | **100** | **0** | **0** | **1** |
| ***fdh*L** | **4463153** | **4464808** |  | **Fructose dehydrogenase large subunit** | **100** | **0** | **0** | **1** |
| ***fdh*C** | **4464805** | **4466223** |  | **Fructose dehydrogenase cytochrome subunit** | **100** | **0** | **0** | **1** |
| ***pgl*** | **4466456** | **4467658** |  | **6-phosphogluconolactonase** | **100** | **0** | **0** | **1** |
| *bes*A | 4471529 | 4472443 |  | Ferri-bacillibactin esterase BesA | 100 | 0 | 0 | 1 |
| *sac*X | 4486743 | 4488113 |  | Negative regulator of SacY activity | 96 | 1 | 8 | 38 |
| *yha*J | 4590574 | 4591470 | c | HTH-type transcriptional regulator YhaJ | 96 | 9 | 12 | 38 |
| *lpo*A | 4593682 | 4595721 |  | Penicillin-binding protein activator LpoA | 94 | 0 | 3 | 40 |
| *htp*X | 4720392 | 4721255 |  | Protease HtpX | 100 | 7 | 9 | 1 |
| *tyr*B | 4722873 | 4724066 | c | Aromatic-amino-acid aminotransferase | 100 | 9 | 10 | 18 |
| *lrp* | 4724146 | 4724619 | c | Leucine-responsive regulatory protein | 100 | 10 | 9 | 20 |
| *fkp*A | 4831399 | 4832223 | c | FKBP-type peptidyl-prolyl cis-trans isomerase FkpA | 98 | 0 | 0 | 67 |
| *ywr*O | 4835600 | 4836151 | c | General stress protein 14 | 100 | 0 | 0 | 46 |
| *mrc*A | 4877346 | 4879904 |  | Penicillin-binding protein 1A | 98 | 11 | 1 | 60 |
| *nud*E | 4879955 | 4880509 | c | ADP compounds hydrolase NudE | 94 | 3 | 1 | 51 |
| *fie*F | 5085392 | 5086294 | c | Ferrous-iron efflux pump FieF | 100 | 4 | 2 | 20 |
