# Supplementary material for: Genomic characterisation of an entomopathogenic strain of Serratia ureilytica in the critically endangered phasmid Dryococelus australis
Source: PLoS One. 2022 Apr 20;17(4):e0265967. doi: 10.1371/journal.pone.0265967 (PMC9020675; doi:10.1371/journal.pone.0265967)
Supplement: S1 Raw images — (PDF) [file pone.0265967.s012.pdf]

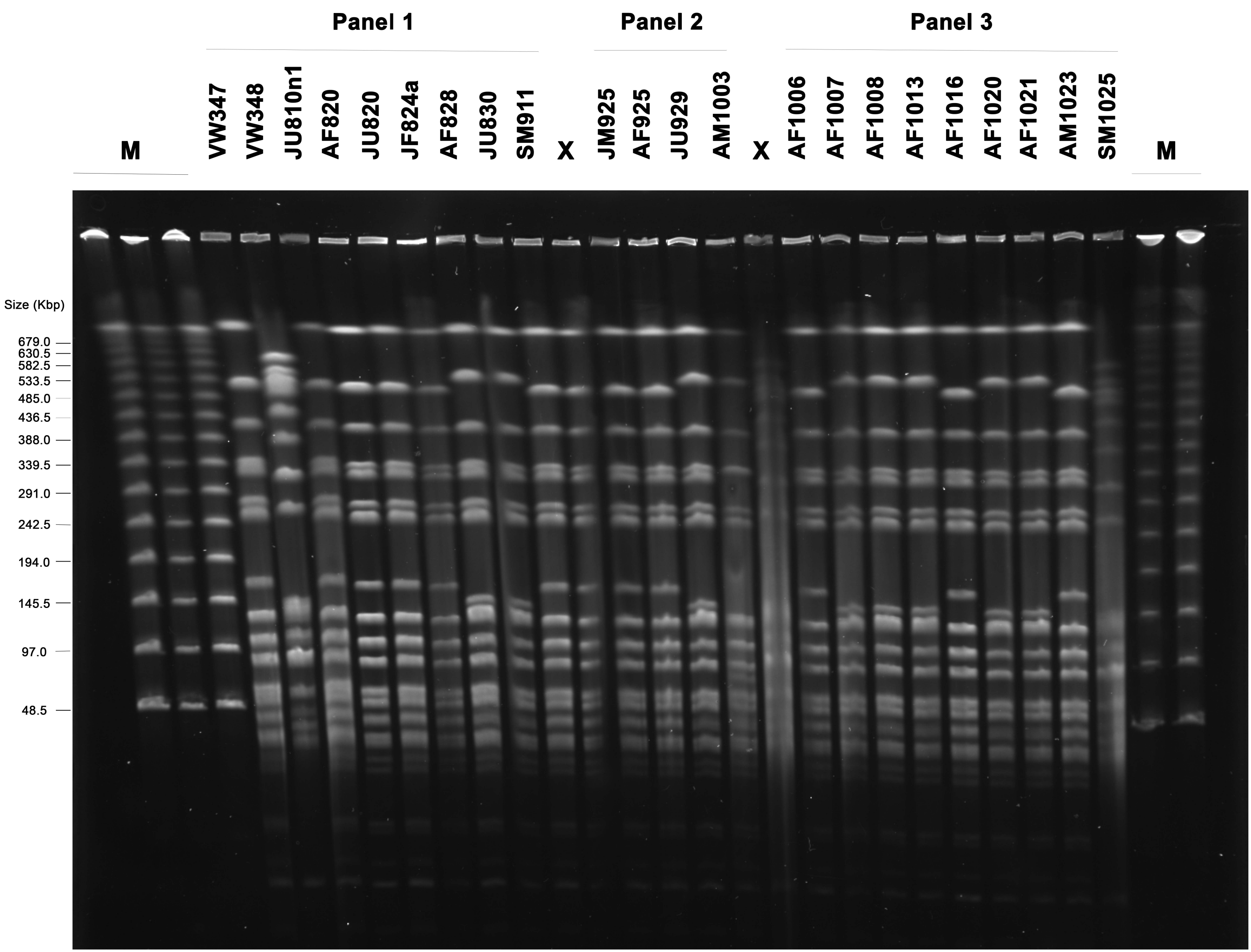

Original image 1, Fig 1:
Method used to capture the image:
Molecular Imager® Gel Doc™ XR System using Image Lab 3.0 software (Bio-Rad Laboratories)

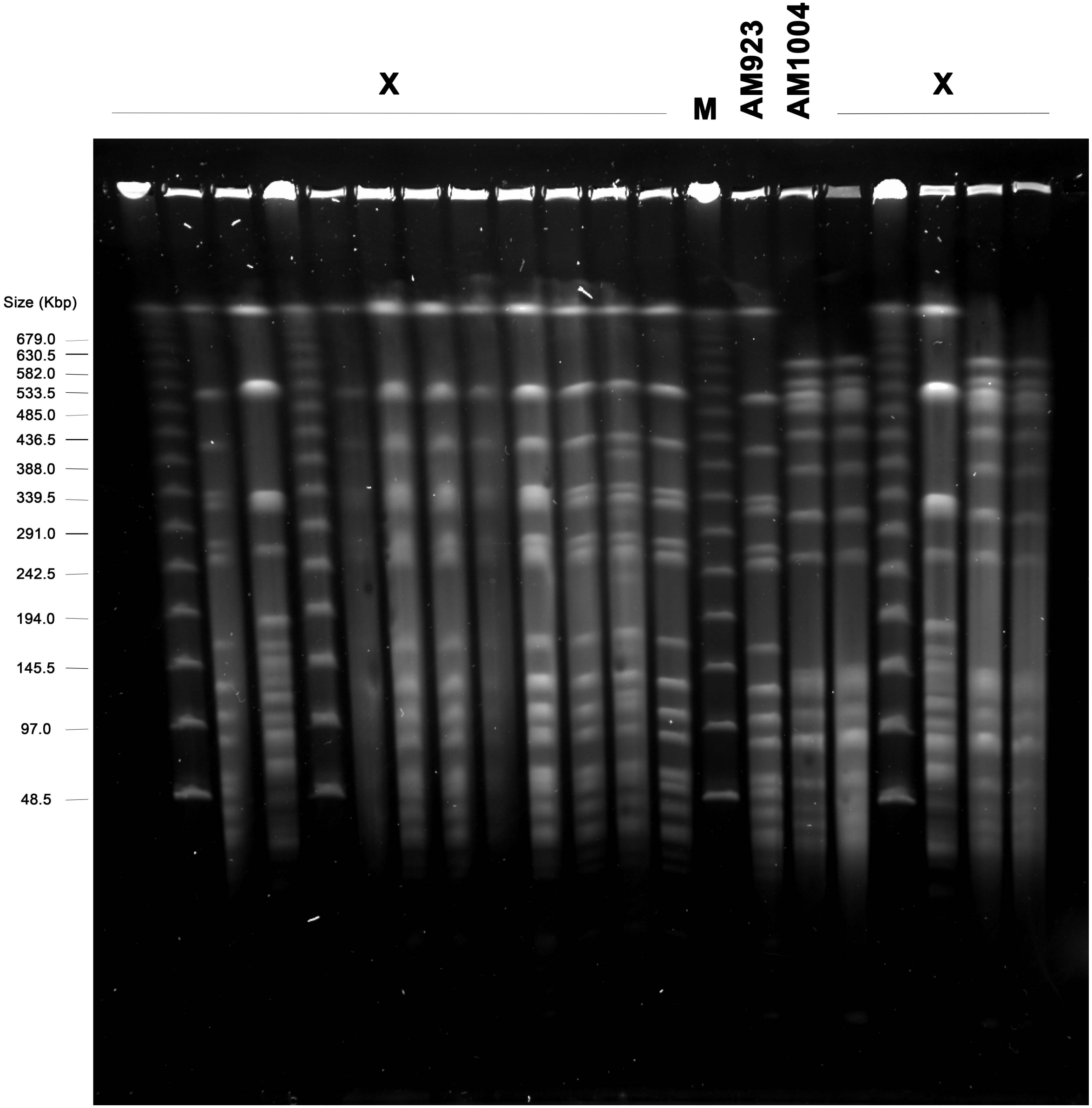

Original image 2, Fig 1:
Method used to capture the image:
Molecular Imager® Gel Doc™ XR System using Image Lab 3.0 software (Bio-Rad Laboratories)

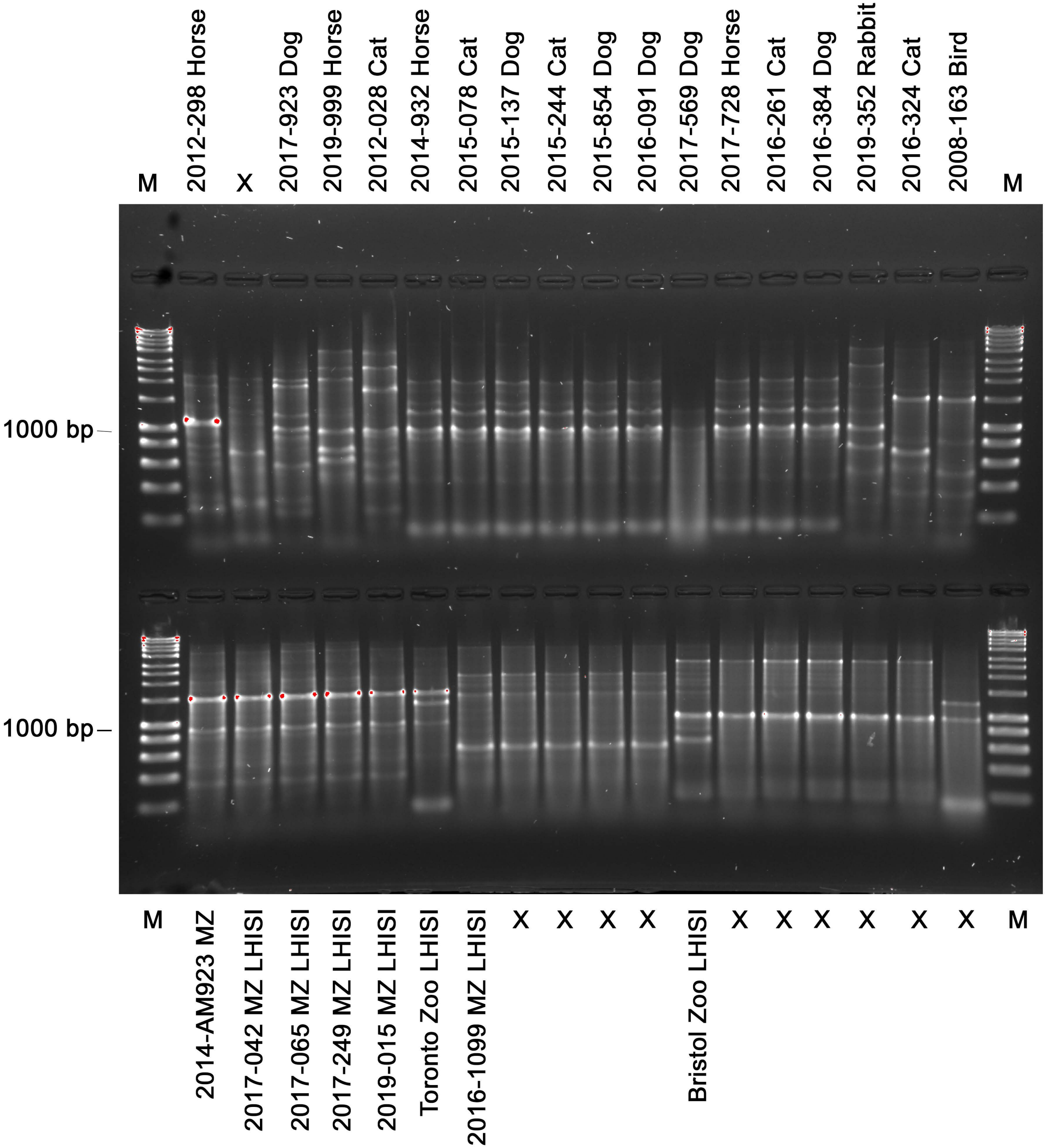

Original image, Fig S2:
Method used to capture the image:
Molecular Imager® Gel Doc™ XR System using Image Lab 3.0 software (Bio-Rad Laboratories)

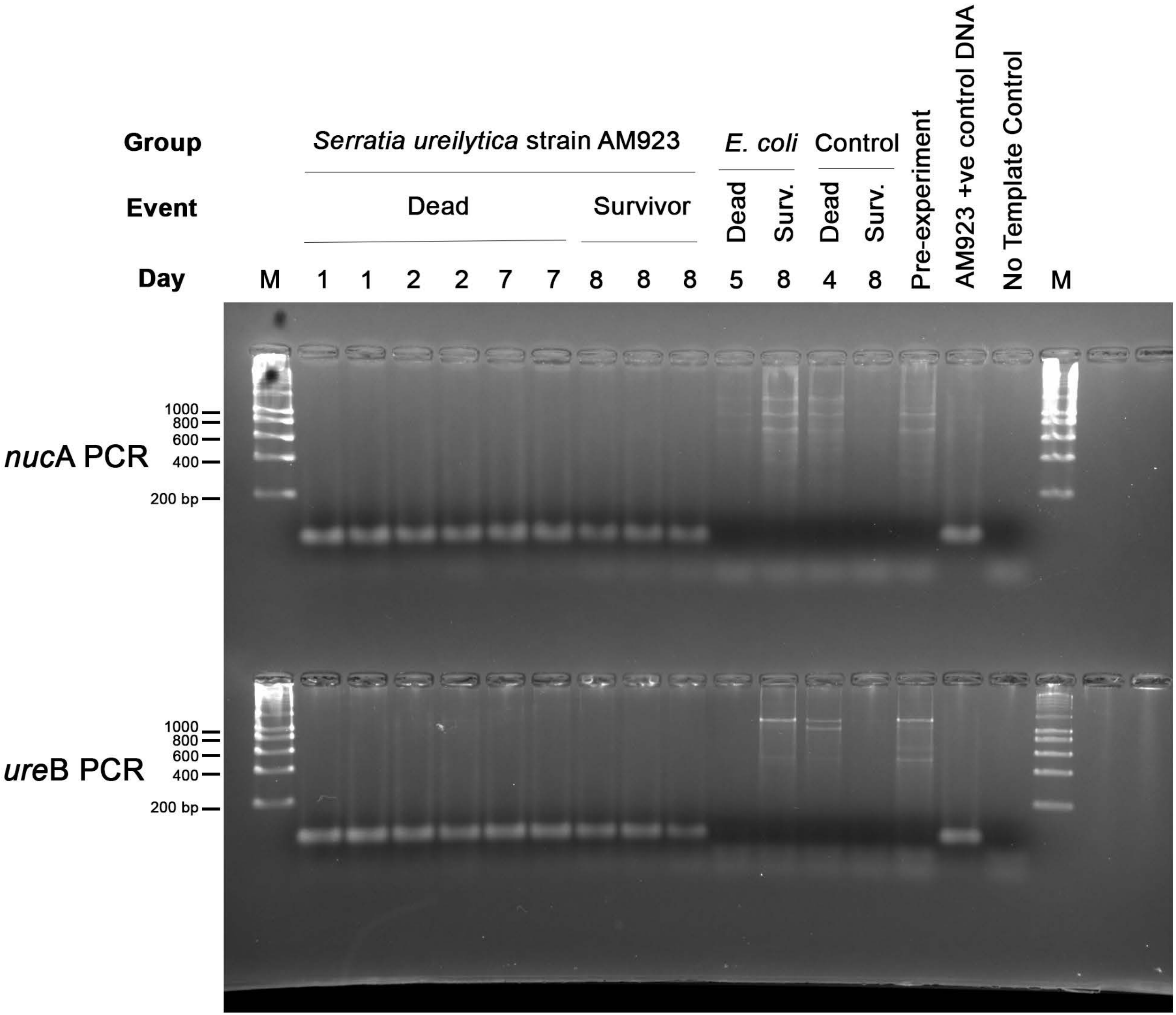

Original image 1, Fig S4:
Method used to capture image: Molecular Imager® Gel Doc™ XR System using Image Lab 3.0 software (Bio-Rad Laboratories)
