# Supplementary material for: Genomic characterisation of an entomopathogenic strain of Serratia ureilytica in the critically endangered phasmid Dryococelus australis
Source: PLoS One. 2022 Apr 20;17(4):e0265967. doi: 10.1371/journal.pone.0265967 (PMC9020675; doi:10.1371/journal.pone.0265967)
Supplement: Attachment — Submitted filename: PlosONE Sureilytica response to reviewers (1).docx [file pone.0265967.s013.docx]

Dear PLOS ONE Editors,

Thank you for providing us with the opportunity of submitting a revised version of our manuscript. We would like to thank the reviewers for their helpful comments and questions. Please find below our response to the reviewer’s remarks and questions.

PONE-D-21-36966: An entomopathogenic strain of *Serratia ureilytica* is associated with mortalities in a captive colony of the critically endangered phasmid *Dryococelus australis*

**Editor’s comments:**

1. Please ensure that your manuscript meets PLOS ONE's style requirements, including those for file naming. The PLOS ONE style templates can be found at 
https://journals.plos.org/plosone/s/file?id=wjVg/PLOSOne_formatting_sample_main_body.pdf and 
<https://journals.plos.org/plosone/s/file?id=ba62/PLOSOne_formatting_sample_title_authors_affiliations.pdf>

We have consulted the PLOS ONE style templates as advised and have made the necessary changes to our manuscript.

2. PLOS ONE now requires that authors provide the original uncropped and unadjusted images underlying all blot or gel results reported in a submission’s figures or Supporting Information files. This policy and the journal’s other requirements for blot/gel reporting and figure preparation are described in detail at https://journals.plos.org/plosone/s/figures#loc-blot-and-gel-reporting-requirements and https://journals.plos.org/plosone/s/figures#loc-preparing-figures-from-image-files. When you submit your revised manuscript, please ensure that your figures adhere fully to these guidelines and provide the original underlying images for all blot or gel data reported in your submission. See the following link for instructions on providing the original image data: <https://journals.plos.org/plosone/s/figures#loc-original-images-for-blots-and-gels>.

We have provided original uncropped and unadjusted images underlying the gel results presented in Figs 1, S1 and S4 uploaded as a supporting information pdf file entitled S1_raw_images.

3. We note that you have included the phrase “data not shown” in your manuscript. Unfortunately, this does not meet our data sharing requirements. PLOS does not permit references to inaccessible data. We require that authors provide all relevant data within the paper, Supporting Information files, or in an acceptable, public repository. Please add a citation to support this phrase or upload the data that corresponds with these findings to a stable repository (such as Figshare or Dryad) and provide and URLs, DOIs, or accession numbers that may be used to access these data. Or, if the data are not a core part of the research being presented in your study, we ask that you remove the phrase that refers to these data.

The data are not a core part of our research presented in this manuscript. Therefore the following statement has been removed from the discussion.

“Attempts to infect *Haemonchus contortus* with AM923 were unsuccessful (JA, pers. comm; data not shown).”

**Reviewer #1 (Matan Shelomi):** My only suggested edit is to define MLST on line 72. Otherwise, the manuscript is excellent, the English level is fluent, and I have no further edits to recommend.

This has been amended in the revised manuscript.

**Reviewer #2:** The title of the study is “ An entomopathogenic strain of Serratia ureilytica is associated with mortalities in a captive colony of the critically endangered phasmid Dryococelus australis”. However, it did not reflect the genetic characterization of the Serratia strains isolated from Dryococelus australis and its phylogenetic relationship with Serratia genus. I suggest that the title be changed to a title that shows all the work done in the present work.

The title has been amended. It now reads:

Genomic characterisation of an entomopathogenic strain of *Serratia ureilytica* in the critically endangered phasmid *Dryococelus australis*

The objectives of this research were: genome sequencing of two representative strains and they were compared with genomes of Serratia spp. Publicly available, including entomopathogenic strains. The virulence of a selected isolate was evaluated in experimentally infected honey bees (Apis mellifera).

The objectives of the study have been rewritten to reflect the reviewer’s comment. The final paragraph of the introduction now reads:

The objectives of this study were: (1) Characterisation of *Serratia* spp. associated with recently deceased and moribund insects of the captive *D. australis* colony of Melbourne Zoo; (2) Determination of the complete genomic sequences of 2 representative strains and comparison with publicly available genomes of *Serratia* spp*.*, including entomopathogenic strains; (3) Assessment of the virulence potential of the predominant isolate in experimentally infected honey bees (*Apis mellifera*).

Hemolymph samples were collected from necropsies of D. australis within the Melbourne Zoo colony that died or were found moribund and euthanized. Serratia, Pseudomonas or Aeromonas and Proteus were isolated from the samples. In addition, environmental samples [nest boxes, floors, drinking water and frass (insect excrement)] were collected.

Molecular typing was performed for Serratia strains based on PFGE and Fingerprinting rep-PCR. For Illumina sequencing, it was performed from two representative strains:: S. ureilytica AM923 and S. marcescens AM1004. The nucleotide sequencing of each strain was analyzed and prophages, genomic islands, antimicrobial resistance genes, and virulence were identified. On the other hand, a phylogenetic and pangenomic analysis was carried out against representative genomes of the genus Serratia downloaded from databases.
The virulence assay was carried out on bees; and finally, the nuclease (nucA) and urease beta subunit (ureB) genes were amplified.

I don't understand why the sequence of strain AM923 was chosen as the main reference genome and an in-depth analysis was performed, while strain AM1004 was used for comparative purposes. In this case, although the genotype of the strain AM923 was the predominant one in relation to AM1004, both genotypes were found in the samples. I think that both strains should be studied with the same interest, for example, both are reported in other Zoo. This means that both genotypes are circulating in the region and it is not known whether they can infect other types of hosts that are equally susceptible to infection by both types of strains.

We chose to study *Serratia ureilytica* AM923 intensively as this genotype has been predominant in the breeding colony since first isolated in 2014 (Fig 1, Fig.2). Despite improvements in the management practices of the LHISI breeding colony, this genotype continues to be isolated from haemolymph of dead and moribund insects (Fig S2). Our phylogenetic analyses show placement of this isolate in clade A, a clade that contains most of the publicly available insect associated *Serratia* spp. genomes.

Based on epidemiological analysis, the genotype represented by AM1004 represents a transient isolate associated with far fewer mortalities within a restricted timeframe. The inclusion of AM1004 demonstrates the complexity in establishing a cause of infection and mortality. However, an equally detailed comparative analysis of both strains would detract the focus of the manuscript from the most significant isolate AM923.

It would also be desirable to perform virulence tests for strain AM1004.

We intend to perform an extensive assessment of virulence, once we gather a collection of the *Serratia* spp. strains from the LHISI colonies. Strain AM1004 will be one of a number of isolates included in a future virulence trial.

Regarding the sequence of the urease operon that could be used for the detection of entomopathogenic strains of Serratia, a statistical analysis is recommended to validate this statement.

The urease PCR detection assay describe in the manuscript was shown to detect strain AM932 as intended. The validation of a detection assay based on this genetic target for broader applications in the field, such as clinical diagnostics, epidemiology and environmental surveillance is beyond the scope of this manuscript. We merely suggest that the urease operon could be the base of such assay due to its distribution in *Serratia* genomes.

The presence of one or two pulsotypes of Serratia strains associated with the infection or death of Dryococelus australis, suggests an epidemic outbreak in the D. australis colony at Melbourne Zoo by one or two strains. It would be important to have the exact date on which the infection occurred in the insects and to relate them to the genetic characteristics of the strains.

Exact dates for haemolymph sample collection have been included within Table S5. Only approximate dates can be established for collection environmental samples and the isolates provided by other zoos.

In addition, to determine the source of infection, it would be good to point out the origin of the environmental samples [nests, floor surfaces, drinking water and frass (insect excrement)] that were contaminated with Serratia, and sequence it, to compare the genome of the Serratia strain isolated from the environmental sample with the genome of the Serratia strain isolated from samples of insect hemolymph.

The origin of the environmental samples with *Serratia* spp. was reported in Table S3. The dendrogram in Fig 2 shows the environmental isolates have pulsotypes that differ from the predominant representative strain, AM923. For this reason, we determined the environmental source of the predominant strain was not identified and further analysis of the genomes from unrelated environmental isolates was not required.

In general, the work is good, however I feel that the main objective is missing, which is the characterization of Serratia spp. strains associated with infection and death of the D. australis colony at Melbourne Zoo.

This has been addressed in an earlier comment.

The authors focused on determining the phylogeny and taxonomy of Serratia genus.
In conclusion, I recommend that the authors rethink the title of the article and the objectives.

This has been addressed in an earlier comment.

**Reviewer #3:** Paper outlines the isolation of insect pathogens and follows the route of species identification using in silico approaches.

To provide context it would be of value to expand on the insect raring conditions, the food source etc and how this in turn may relate to their natural habitat. From here can then associate with Serratia species

Reference to Carlile et al. (2009) that details the history and raring conditions has been made in the introduction.

Though I see the rational for the use of Bees as a proxy for stick insects, which in turn may explain the use of 37 Celsius microbial growth parameters. Assessing an insect from the same family might be more preferable, more so as many insect pathogens are host specific

We agree with the reviewer’s opinion that a Phasmid may constitute an excellent alternative for developing a more closely related infection model. *Serratia* spp. are commonly present within species of Phasmid and previously we have found it difficult to confirm *Serratia-*free status of insects allowing for an informative virulence assay, but we hope that the development of improved diagnostic assays will allow for this in the future.

We have stated that we have only partially fulfilled Koch’s postulates in using the honey bee infection model and reported the “virulence potential” of *Serratia ureilytica* AM923 in the Lord Howe Island stick insect. Use of the honey bee infection model enabled comparison with other entomopathogenic isolates of *Serratia* spp. established by Raymann et al.(2018).

In relation to the disease are there any photos of the diseased insect. I note histology mentioned L103, some images would be of value - where is this data .
Table S4 lists observed plaques wherein some photos would help or for that matter more detailed bioassay data, host range and dose response data would be of value

Images of gross pathology and histopathology are featured in Bayley et al. 2018, referenced in the introduction and materials and methods.

In general greater detail could be provided in Figure legends

We have reviewed all figure legends.

The following changes have been made:

- Figure 6: The confidence interval (95%) has been included
- Figure S1. See specific comment below
- Figures S2 and S4. Details of the molecular weight marker have been included.

I feel that as written the manuscript is too focused on use of in silico approaches to define species and needs to be bolstered in mode of action and bioassay data more so to provide relevance to the study and for that matter the phasmid. In silico analysis can then assess for presence of insect active enzymes/toxins etc .

The *Serratia* isolates were characterised by conventional microbiology and genotyping methods. However, it was essential to use extensive genome sequence data analysis to define and classify the species correctly within this genus.

According to Grimont et al. 2006, urease production is a variable characteristic for *Serratia* spp. with most testing negative. Initially, our protocol for the identification of *Serratia* spp. did not include urease production.

Once it was established that the predominant isolate was urease positive, our identification protocol for isolates cultured from the virulence assay was adjusted to include the biochemical test for urease production.

## We have amended Figure S1 and legend to now include results of the phenotypic test for urease production. Mention of these results in the section entitled: Development of PCR assays for detection of entomopathogenic *Serratia* spp*.* based on genome analysis.

In addition to this more information on how this study could help with raring phasmids in the discussion would be of value

This has been addressed in an earlier comment.

Virulence assay
I am left unsure on the definition of carriage or a timeframe (line 465 denotes 6 days), I am left asking several questions :-

The sentence referring to carriage now reads:

To enable screening for the presence of *Serratia* spp., randomly selected bees were removed and euthanised by chilling to -80°C prior to the experiment.

The timeframe (8 days) has been added to the Material and Methods section. The sentence now reads:

After a period of 8 days, the surviving bees were counted and euthanised by chilling to -80˚C.

were they surface sterilised prior to isolation

The insects were not sterilised prior to isolation on MacConkey agar. Surface sterilisation of tissues would not have been practical in this study and may have caused failure of the bacteriological isolation procedure. Unfortunately, we cannot eliminate the possibility of detecting *Serratia* spp. on the surface of the bees. However, based on the differing amount of *Serratia* spp. cultured from dead bees compared to those that survived the experiment, we are confident we have shown AM923 is entomopathogenic in bees.

was a dilution series undertaken prior platting

A dilution series was not undertaken prior to plating on MacConkey agar. However, the streak-dilution method used to plate the samples conveys a semi-quantitative element of information.

what is the dominance of the isolated bacteria relative to the other isolated bacteria -if any.

We used MacConkey agar isolate *Serratia ureilytica* AM923 from bees at the completion of the experiment. We chose not to use a rich non-selective agar for this procedure to minimise the growth of other bacteria, should other bacterial species be present. For this reason, we do not have the data to address this question.

In this context Table 1 lists some numbers. In this respect n=127 does this mean colonies total from an insect ? or something else - please clarify and provide a quantifiable measure , a foot note on definition of pure and mixed would be useful

127 reflects the number of haemolymph samples collected from Lord Howe Island stick insects that were cultured. It is not common practice in clinical diagnostic microbiological investigations to enumerate bacterial colonies, instead the number of different colony types is recorded and the amount of growth described eg. heavy, moderate and light. It is for this reason that the number of colonies cultured from each sample was not determined.

Pure cultures refer to cultures where a single colony type is growing on the agar plate, whereas a mixed culture has any number of different colony types.

In considering this comment, we have revised Table 1 to remove duplication and improve clarity by separating data related to pure and mixed cultures. A statement about the numbers of negative samples was add to the paragraph, previously it had been included in Table 1.

L211 I am unsure of this assay it read as a direct immersion of a bee in a solution of bacteria based on the provide information it is hard to know how the bacteria gets ingested via this technique , perhaps using a sucrose bacteria solution applied to a dental wick might be more apt

We have referenced Raymann et al. (2018) that describes the technique used to inoculate the bees in more depth and the details of all modifications to the published method are stated in the Materials and Methods section.

Kochs postulates i.e. would be nice to see i.e. re feed the suspected bacteria back to the insect species it was isolated from to see affect again . I appreciate a rare insect so perhaps a closely related species might act as a more suitable proxy than the honey bee

In addition it appears the bacteria were reisolated and validated through PCR were the insects surface sterilised prior to this step?? I am mainly concerned with PCR sensitivity and its ability to differentiate live verse dead (DNA) cells . In this context platting out is a likely more applicable method more so if we assume that a pathogen might multiply and dominate the microbial population. It would be good to have a quantifiable measure eg from each cadaver ## bacteria were isolated

Re feeding the isolated bacteria to a new batch of insects and enumerating bacteria recovered from dead and surviving bees are good suggestions and improvements that we could consider for future experimental trials.

We have used PCR targeting the *ure*B gene to detect *Serratia ureilytica* AM923 and direct culture of crushed abdomens of bees on MacConkey agar as alternative approaches to partially fulfil Koch’s postulates. As our analyses have shown, presence of the urease operon is mainly restricted to clade A, where many isolates have been described as entomopathogenic. The PCR was used successfully to detected *Serratia* sp. in the dead and surviving bees of the group treated with AM923, but not the Groups exposed to *E. coli* K-12 and the negative control. We have also described differences in the amount of growth on MacConkey between bees that died and those that survived the trial. A heavy culture of *Serratia* sp. grew from dead bees compared to light growth on plates cultured from surviving insects within the treatment group. There was no growth of *Serratia* spp. in cultures from the *E. coli* K-12 treatment group, the negative control group and bees collected prior to the virulence assay. We are satisfied that we have reisolated AM923 in significantly higher numbers from dead honey bees treated with *Serratia ureilytica* AM923.

Phylogenies are not my expertise , this study are restricted based on species where sequenced genomes are available, this is okay but I think a MLST /16S encompassing all Serratia species (Type strains – of which data is available ) would help complement the extensive genome based phylogenies and help the reader better understand where the Serratia strain fits and of course interpretation of the data.

The type strains, based on information available on the LPSN website, have been highlighted in Tables S1 and S2.

A MLST scheme has not been developed for *Serratia* spp. and sequencing of the 16s rRNA gene does not provide the level of discriminatory power required for characterisation of this species. For these reasons, we have undertaken full genomic sequencing that enables comparison of the predominant isolate with other published *Serratia* spp. genomes.

We have included the following statement in the introduction in response to this comment:… sequencing of the 16s rRNA gene does not provide sufficient discriminatory power to characterise isolates.

The authors also allude to the role of chitinases etc L559 in other systems but I am unsure if they assessed this in the genomes sequenced in this study. L553-560 are pertinent points and leaves the reader wondering what accessory virulence determinants are in the assessed Serratia genome - in this context a preliminary genome assessment of Haemolysin chitinase etc would be of value . This is possibly more relevant than Urease

We chose to focus our investigations on the urease operon for the following reasons:

- Only a small percentage of *Serratia* spp. have been found to produce urease.
- Phenotypic testing of the LHISI isolates showed they were positive for urease production after 48 hours incubation.
- Whole genome sequencing of both AM923 and AM1004 determined the urease operon was complete
- clade A contained most of the insect associated isolates and all possessed the urease operon
- Homology exists between the urease operon of all insect associated *Serratia* spp. isolates sequenced thus far
- Phylogenetic analysis that compared our isolates with publicly available genome sequences showed clade A (the insect associated clade) had 100% carriage of the urease operon, whereas clades B and C showed the less that 20% (Data shown in Figure S7)

We have made mention in the discussion of other possible virulence factors as referenced in other studies. All novel genomic sequences generated within this project will be made publicly available and open for further analysis by others. Documenting the carriage of all possible virulence factors was not the aim of this was paper.

Fig S2 is nice given a finite set of Serratia species (due to genome access limitations), please reference genome accession numbers. This is also applicable for other figures and Tables (I might have missed this information)

The genome accession numbers and references are provided in Table S1.

Of interest in the discussion the authors acknowledge and discuss many of the issues I have raised such as use of bee model etc

Minor comments are as per below
L37 Based on in silico analysis ? a urease operon …..

Phenotypic testing was also conducted that confirms the urease is present and functional.

The phenotypic urease test results have been included in Fig S2 and a statement in the results section reads:

The presence of urease activity in strain AM923 and other strains positive in the *ure*B PCR assay was confirmed by phenotypic testing, which detected a positive reaction on urea agar slopes after 48 hours of incubation at 37˚C (Fig S2).

L52 could list food source

The following reference has been included to provide more details of the food sources and other information about management of the phasmids within this breeding colony.

Carlile N, Priddel D, Honan P. The recovery programme for the Lord Howe Island Phasmid (*Dryocoelus australis*) following its rediscovery. Ecol Manag Restor. 2009; 10:S124-S128.

L57 is bacilli the correct term please check is this relating to Serratia? might be rods? I am unsure

This has been amended in the revised manuscript. Bacilli replaced by rods.

L61 -62 why have we singled out these the Serratia specie?

Our focus on *Serratia* spp. stems from the high proportion (> 80%) of LHISI haemolymph samples positive for *Serratia* spp. reported by Bayley et al, 2018. Of the 127 samples analysed in this study, *Serratia* spp. was isolated in pure culture from 71.2% of samples.

We have included this statement in the introduction to highlight the significance of these findings:

Haemolymph is a normally sterile site and pure growth of bacteria in high cell numbers on a rich non-selective culture medium, such as sheep blood agar, is deemed to be clinically important.

L102 rational for using SBA and MAC?

The combination of SBA/MAC at 37˚C is the standard protocol practiced by most diagnostic laboratories for isolation of bacterial species from clinical specimens and samples. SBA is a rich non-selective medium on which most cultivable bacteria can grow. MacConkey agar is selective for non-fastidious organisms that can survive the presence of bile salts. This usually means Gram negative, enteric bacteria but some Gram positive bacteria such as staphylococci are also able to grow. Culture on MacConkey provides information about the fastidious nature and ability of the isolates to ferment lactose.

Latter L118 LB then L130 TSB then used? I note MAC is used to selectively isolate Gram-negative and enteric bacteria

LB and TSB are non-selective media and can support the growth of *Serratia* spp. LB agar containing cycloheximide was chosen for the detection of *Serratia* spp. within environmental samples to minimise the occurrence of other contaminants, such as fungi.

The richer TSB broth medium was used to cultivate purified isolates of *Serratia* spp. for the starting material used for DNA extraction and genomic analysis.

L103 is there a rational for 37C just curious might be of interest to understand the ambient temperature the phasmids insects are in

This is addressed in a previous comment.

L118 perhaps mention cycloheximide is an anti fungal

This has been amended in the revised manuscript.

L120 could get the 16s of the bacteria sequenced - in addition is there a predominant isolate

We chose to use a biochemical approach to initially identify *Serratia* spp. rather than of 16s rRNA sequencing. The results of the pulsed field gel electrophoresis (Fig 1) and rep-PCR (Fig S2, previously Fig S1) suggested there was a predominant isolate, evidenced by the consistency of the banding patterns produced by restriction endonuclease digestion with SpeI, a rare cutting restriction endonuclease.

Figure 6 Time? I assume days post challenge please provide value, I am unsure what number at risk relates to.

Figure 6 has been amended. Time on the x axes has been replaced by Days post challenge and the heading for the second graph now reads Number of insects at risk.

L212 full genus for E coli

This has been amended in the revised manuscript.

L213 has PBS been written in full and components provided?

This has been amended in the revised manuscript.

Table S7 I am unsure of the relevance of this Table

Table S7 lists the CDSs in all insect isolates from clade A that are present in fewer than 20% of isolates from clades B and C. The table is relevant to this study as it provides detail and more evidence of the relationship of genomes within clade A and contrasting with those with clades B and C. It supports our approach to focus the development of a specific diagnostic tool based on the urease operon to detect the predominant *Serratia ureilytica* strain within the LHISI.

L223 what is a cup cage perhaps dimensions could be provided?

A cup cage was used to contain bees during the challenge experiment. We have added a new figure with images of the cup cage in the supplementary material (Fig S1).

L319 could list the program used prior to … Using #### the chromosome was predicted

Details of the tools to predict prophages and integrative conjugation elements (ICEs) have been added to the materials and methods section.

L489 what is defined as a harsh environment

We have provided references that describe the harsh or extreme environments from which *Serratia ureilytica* has been isolated.

Filippidou et al. (2019) describes the harsh conditions of Lirima, a geothermal site in the Andean highlands of northern Chile, where *Serratia ureilytica* Lr5/4 was isolated. Environmental stressors present at this site include high levels of UV radiation, variable daily temperatures, high altitude and low relative humidity.

We have substituted harsh with extreme.

Fig S1 mention why a subset of isolates are red hashed boxed. i.e. red hashed boxed denotes Lord H..

The following sentence has been added to the figure legend of Fig S2 (previously Fig S1):

A subset of Lord Howe Island stick insect isolates from Melbourne Zoo in 2017 and 2019, indicated by the red hashed box, displayed a highly similar rep-PCR fingerprint to *Serratia ureilytica* AM923.

6. PLOS authors have the option to publish the peer review history of their article ([what does this mean?](https://journals.plos.org/plosone/s/editorial-and-peer-review-process#loc-peer-review-history)). If published, this will include your full peer review and any attached files.
