# Supplementary material for: Genomic characterisation of an entomopathogenic strain of Serratia ureilytica in the critically endangered phasmid Dryococelus australis
Source: PLoS One. 2022 Apr 20;17(4):e0265967. doi: 10.1371/journal.pone.0265967 (PMC9020675; doi:10.1371/journal.pone.0265967)
Supplement: Attachment — Submitted filename: Response to reviewers submission 2.docx [file pone.0265967.s014.docx]

Dear PLOS ONE Editors,

Thank you for providing us with the opportunity to submit a revised version of our manuscript.

PONE-D-21-36966: An entomopathogenic strain of *Serratia ureilytica* is associated with mortalities in a captive colony of the critically endangered phasmid *Dryococelus australis*

Reviewer #3: The paper is much improved, specifically the introduction and the results, however the discussion lacks a clear focus and highlighting the outcomes of the study --and could be reworked . The responces in places are good but I might suggest that they accordingly update the manuscript with the information in the response provided

A photo of a diseased insect referenced in Bayley however I am left unsure if isolates from the Bayley paper were included in this study? if so please highlight this, if not this would further warrant the inclusion of a photo/histology from where it could be compared to that of Bayley et al

Bayley *et al.* 2018 provides a description of the disease presentation within insects of the same colony. The histopathological analysis reported in this paper was also conducted by the same authors.

We feel inclusion of an additional figure is likely to detract from the key microbiological findings of this paper.

L120 I am still left unsure of what insects are feeding on - what is the plant material is it fresh a live plant etc??

The following information has been included:

Plant material refers to potted plants or fresh clippings of tree lucerne or tagasaste (*Chamaecytisus prolifer*), Lord Howe Island tea tree (*Melaleuka howeana*), Morton Bay fig (*Ficus macrophylla*), holly oak (*Quercus ilex*), *Baloghia* sp. and *Pittosporum* sp. provided as a food source.

L125 right LB in full first time mentioned

LB is in common usage and refers to a standard culture medium. There is confusion about what LB stands for. This medium has previously been referred to as Luria broth, Luria-Bertani medium, Lennox broth and Lysogeny broth and we think using any of these other names would create confusion for readers.

L280 perhaps put a descriptor on what the other groups are

A description of other bacteria isolated from haemolymph samples has been provided in Materials and Methods, Section: Bacterial isolation and identification

L343 344 list % ANI value after each species name in brackets

This information has been added, despite this information being already available in S6 Table.

L464 for future reference you could incorporate through primer synthesis in generic primer base at points of difference e.g. a N or a Y etc

Thank you, this point has been noted. No change has been made to the manuscript.

L497 what is the extreme variable salinity , temp or other?? please update as per response

This information was not determined by the experiments featured in this manuscript. The referenced journal article describes the extreme environment from which *S. ureilytica* has been isolated previously. The reviewer has requested that we provide information that is not within our remit and its omission does not detract from the key findings of this paper.

Discussion -reads as disjointed and seems to focus on negatives detracting from the reading. I believe a discussion should be written in a positive context and highlighting key results

After careful examination of the discussion, we believe that the text provides a balanced view of our study, and adequately represents the novelty and significance of the results while avoiding overreaching conclusions.

L503 do we mean isolated as a pure culture ?

We have replaced the word “grown” with isolated.

….L504-505 could be deleted as detracts from the prior sentence

This sentence has been deleted.

L505-506 are there pictures of the histology and I would possibly shift sentence to latter on after you have discussed the bacteria

This comment has been addressed previously.

L514 paragraph. I still think we need to be quantitative did Pseud and Prot dominate the % microbes isolated from these insects

This information is difficult to measure accurately by visual inspection of standard non-selective agar plates that have been inoculated by the streak-dilution method. While this technique can provide semi-quantitative data in the case of a pure growth of an organism from a clinical specimen collected from a mono-bacterial infection, it is not sufficiently precise to evaluate the relative proportions of different bacterial species in a mixed population. We therefore cannot provide reliable data to address this question.

L532 clarify who submitted the sequences to NCBI , if they -are from this study the sentence can be removed and leave it to NCBI to reassign the species , please clarify

We have replaced used with “included in”.

L533-L539 may irk some readers could reduce text here and often good to cross reference/compare to a type strains, we can assume there is a lot of rubbish in the NCBI database,

Where genomic sequence is publicly available, type strains have been included in our analyses. We have highlighted the type strains used in this study, in response to a previous comment from this reviewer in the first review. (S1 and S2 figures)

L534 what do we mean by reinterpreted I might have suggested that these strains be included further strengthening the article

L541 this paragraph seems to have no value , what is its context and how does it relate to the results in this study

The comments L532, L533-L539, L534, L541 all relate to a single paragraph and our response to all is below:

The *Serratia* spp. sequences listed in S1 Table were submitted to NCBI by numerous parties. The only sequences related to this paper, submitted by the authors were *S. ureilytica* AM923 and *S. marcescens* AM1004. The species was assigned by NCBI for all genome sequences submitted by the authors.

Our observation that we have attempted to document in this paragraph is the *Serratia* genus phylogeny is complex and requires some attention from NCBI to redress the assignment of species. This is currently ongoing through the systematic re-assessment of the metadata of Serratia genomes deposited in the GenBank database. A “taxonomy check” is now provided to compare the “declared organism”, the “Best-matching type-strain assembly for declared species” and the “Best-matching type-strain assembly”. A number of genomes originally declared as *S. marcescens* and used in this study are now reported as having failed the taxonomy check. We expect that the taxonomic organisation of the genus will become gradually more stable in the future as species are better defined. For the time being, *S. marcescens sensu lato,* as a term, was suggested to encompass genomes within clade A that are mainly associated with insects, sharing a high homology to the urease positive *S. ureilytica* AM923, despite other species names assigned to genomes submitted at a time prior to the elucidation of the full genomic sequence of *S. ureilytica*.

L606 what are these genomic targets

The genomic targets identified by this research are *ure*B and other 6 genes of the urease operon, nickel/cobalt transporter gene (*hoxN*) and 6-phosphogluconolactonase gene (*plg*). As shown in the AM923 (Fig 5.) and described in L424 – L433.
